# *In Situ* Multiscale Imaging Reveals Cellular Selection and Remodeling of Peptide Assemblies from Membrane Perturbation to Actin Dysregulation

**DOI:** 10.1021/jacs.6c14737

**Published:** 2026-09-10

**Authors:** Jiaqi Guo, Ayisha Zia, Wangbiao Guo, Shoichi Tachiyama, Kangqiang Qiu, Jiaqi Wang, Jack Botting, Zhiyu Liu, Yuchen Qiao, Jiajie Diao, Jun Liu, Fengbin Wang, Bing Xu

**Affiliations:** † Department of Chemistry, 8244 Brandeis University, Waltham, Massachusetts 02453, United States; ‡ Department of Biochemistry and Molecular Genetics, 9967 University of Alabama at Birmingham, Birmingham, Alabama 35294, United States; § Department of Microbial Pathogenesis, Yale School of Medicine, New Haven, Connecticut 06536, United States; ∥ Microbial Sciences Institute, 5755 Yale University, West Haven, Connecticut 06516, United States; ⊥ O’Neal Comprehensive Cancer Center University of Alabama at Birmingham, Birmingham, Alabama 35294, United States; # Department of Cancer Biology, Center for Chemical Imaging in Biomedicine, Advanced Cell Analysis Service Center, University of Cincinnati College of Medicine, Cincinnati, Ohio 45267, United States

## Abstract

Enzyme-responsive peptide nanomaterials can induce non-apoptotic cancer cell death, yet the supramolecular states that mediate activity in cellular environments are usually inferred rather than directly resolved. Here, we integrate multiscale *in situ* imaging with biochemical assays to map distinct assembly states of the phosphatase-responsive peptide NBD-L4_p_Y across cell-free and cellular environments. Cell-free cryo-electron microscopy (cryo-EM) reveals concentration- and history-dependent cross-β polymorphs. An equilibrated NBD-L4_p_Y solution shows no detectable filamentous assemblies, whereas rapid dilution from a higher concentration produces a mixed filament population containing a ∼7 nm C13 nanotube. At the cell interface, this accessible C13 packing architecture becomes predominant and is resolved *in situ* at 3.4 Å. Cryo-electron tomography (cryo-ET) places these nanotubes to membrane contact and invagination sites associated with localized membrane perturbation and early permeability changes. At later times, cryo-ET of lamellae prepared by cryo-focused ion beam (cryo-FIB) milling reveals morphologically distinct intracellular assemblies associated with filamentous actin and membranous organelles. Fractionation shows profilin-1 (PFN1) redistribution into peptide-rich pellets, whereas reconstitution demonstrates dose-dependent impairment of actin polymerization. Together, the data support a two-stage model in which cellular environments first favor a membrane-active nanotube and subsequently promote supramolecular remodeling into morphologically distinct intracellular assemblies associated with altered actin regulation. Thus, context-dependent structure selection and remodeling, rather than enzyme activation alone, emerge as key determinants of bioactive supramolecular materials.

## Graphical abstract

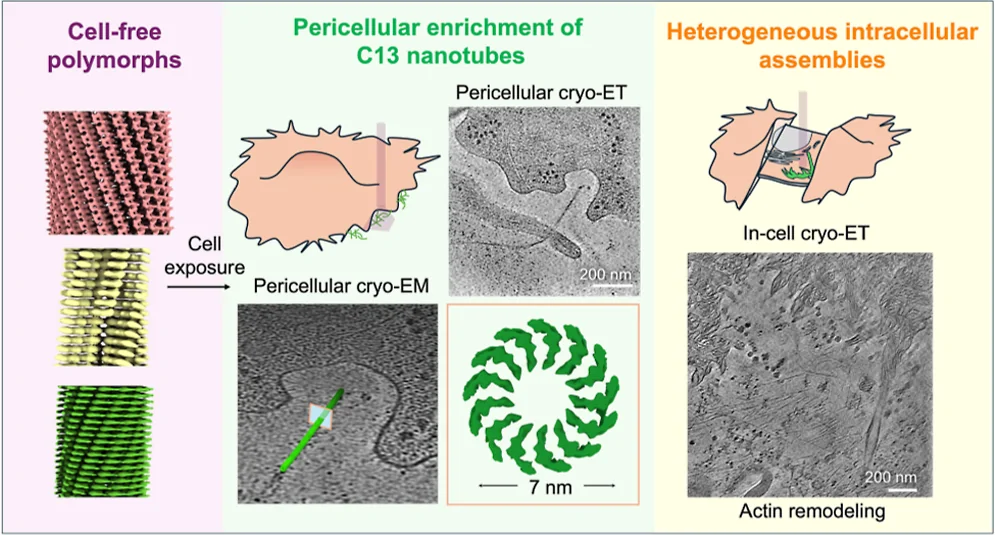

## Introduction

Synthetic nanostructures that assemble in living systems offer a powerful tool to reprogram biological function with spatial and temporal precision. Among these, supramolecular peptide nanostructures are particularly attractive because subtle changes^1–10^ in the local cellular environment, such as enzymatic activity,^4–7^ can trigger large structural transitions that reshape cellular behavior. Despite rapid progress in designing responsive peptide nanomaterials, a central challenge remains: which specific supramolecular nanostructures arise within the complex cellular milieu, and how these dynamic nanostructures disrupt cellular organization and function. Thus, the key question is not only whether enzyme-instructed peptide assemblies form in cells, but which supramolecular structures are selected by the cellular environment and how those structures execute biological function.

This challenge is particularly acute for enzyme-instructed self-assembly (EISA). Enzymatic conversion generates spatial gradients and transient local enrichment that bias nucleation and growth, potentially favoring assembly states that are different from bulk, equilibrated samples. In cells, these assemblies can encounter the extracellular matrix, membranes, cytoskeletons, and organelles that further reshape the assembly profile. Disentangling these effects requires not only identifying the supramolecular nanostructures, but also placing them in their native cellular context and tracking how they evolve over time. Based on this context-dependence, peptide building blocks can adopt distinct supramolecular states depending on local concentration, conversion kinetics, and microenvironment, which have been explored to regulate cell fate,^11–13^ kill tumor cells,^14–18^ inhibit bacterial growth,^19–22^ and guide tissue regeneration.^11,23,24^

Cryo-EM^25^ has emerged as a powerful approach to determine peptide self-assembly architectures *in vitro* at high resolution,^6,12,26–31^ whereas fluorescence microscopy maps their intracellular distributions and dynamics at the whole-cell level.^7,32^ However, the atomic structures of peptide assemblies in the cellular milieu and their direct physical interfaces with membranes and cytoskeletal components remain largely unexplored.

A recent study of an alkaline-phosphatase-responsive phosphopeptide resolved multiple nanotube polymorphs at high resolution in cell-free solutions and used cryo-ET to visualize filament penetration of the plasma membrane, thereby linking ordered supramolecular packing to mechanical membrane failure and extrinsic lytic death.^33^ However, because the membrane-engaged filaments were not themselves reconstructed at near-atomic resolution in the cellular environment, it remained unclear whether the observed cellular function was executed collectively by the polymorphic ensemble or predominantly by a particular polymorph selected at the cell interface. This distinction cannot be resolved from filament diameter or gross morphology alone, because related assemblies may slightly differ in symmetry, molecular packing, and biological interactions. Here we address this gap using a multiscale *in situ* imaging and biochemical workflow. We focus on NBD-L4_p_Y, an enzyme-responsive peptide previously shown to selectively kill human induced pluripotent stem cells and osteosarcoma cells through non-apoptotic mechanisms.^34,35^ We first define the concentration- and history-dependent assembly landscape of NBD-L4_p_Y in cell-free conditions, revealing a polymorphic ensemble of candidate supramolecular states. The ∼7 nm C13 nanotube is transiently accessible after rapid dilution *in vitro* but is not detected in the equilibrated 400 μM solution. At the cell interface, this architecture becomes predominant and is resolved *in situ* at 3.4 Å. Cryo-ET places these pericellular assemblies at membrane-contact and invagination sites associated with localized membrane perturbation and peptide entry. After entry, the peptide remodels into dense intracellular assemblies that interface with filamentous actin (F-actin) and membranous organelles. Finally, biochemical assays show selective enrichment of PFN1 in peptide deposits and direct inhibition of actin polymerization.

By integrating structural determination with spatiotemporal mapping and molecular validation,^36^ our results reveal a two-stage mechanism. Enzymatic peptide assembly first generates a structurally defined nanotube that is associated with membrane perturbation and subsequently reorganizes into dense intracellular assemblies that disrupt actin regulation. These findings establish a direct structural link between context-dependent supramolecular assembly and cellular dysfunction. More broadly, this work shows how enzymatic cues and cellular environments select specific nanostructures and how these nanostructures actively remodel higher-order protein assemblies within living cells.^37^ It also offers insight into reaction-diffusion processes at micro- and nanoscales,^38^ and provides design principles for responsive nanomaterials that operate across biological length scales.

## Results and Discussion

### NBD-L4_p_Y Exhibits Concentration- and History-dependent Self-Assembly

The peptide NBD-L4_p_Y consists of three parts: (i) 7-nitrobenzofurazan (NBD) that serves as an aggregation-sensitive fluorescent probe to report cellular distribution of assemblies;^32,39^ (ii) a hydrophobic and rigid tetra-leucine (L4) motif to promote self-assembly; and (iii) a C-terminal phosphotyrosine (_p_Y) to introduce amphiphilicity and phosphatase-responsiveness (Figure 1A). As previously described,^34,35^ we synthesized NBD-L4_p_Y through solid-phase peptide synthesis, purified via high-performance liquid chromatography (HPLC), and confirmed its identity via liquid chromatography–mass spectrometry (LC–MS) (Figure S1). In the presence of alkaline phosphatase (ALP), NBD-L4_p_Y underwent near-complete dephosphorylation to form NBD-L4Y (Figure S2). This process triggered self-assembly, producing distinct nanostructures depending on the starting concentration. Specifically, at 400 μM, particles transformed into ribbons, whereas at 10 mM, nanotubes transformed into curved morphologies (Figure 1B). A similar concentration-dependent morphogenesis was observed for the enantiomer (D)-NBD-l4_p_y, with a gradual transition from ribbons to truncated or broken ribbons, and eventually to curvatures (Figure S3).

**Figure 1:**
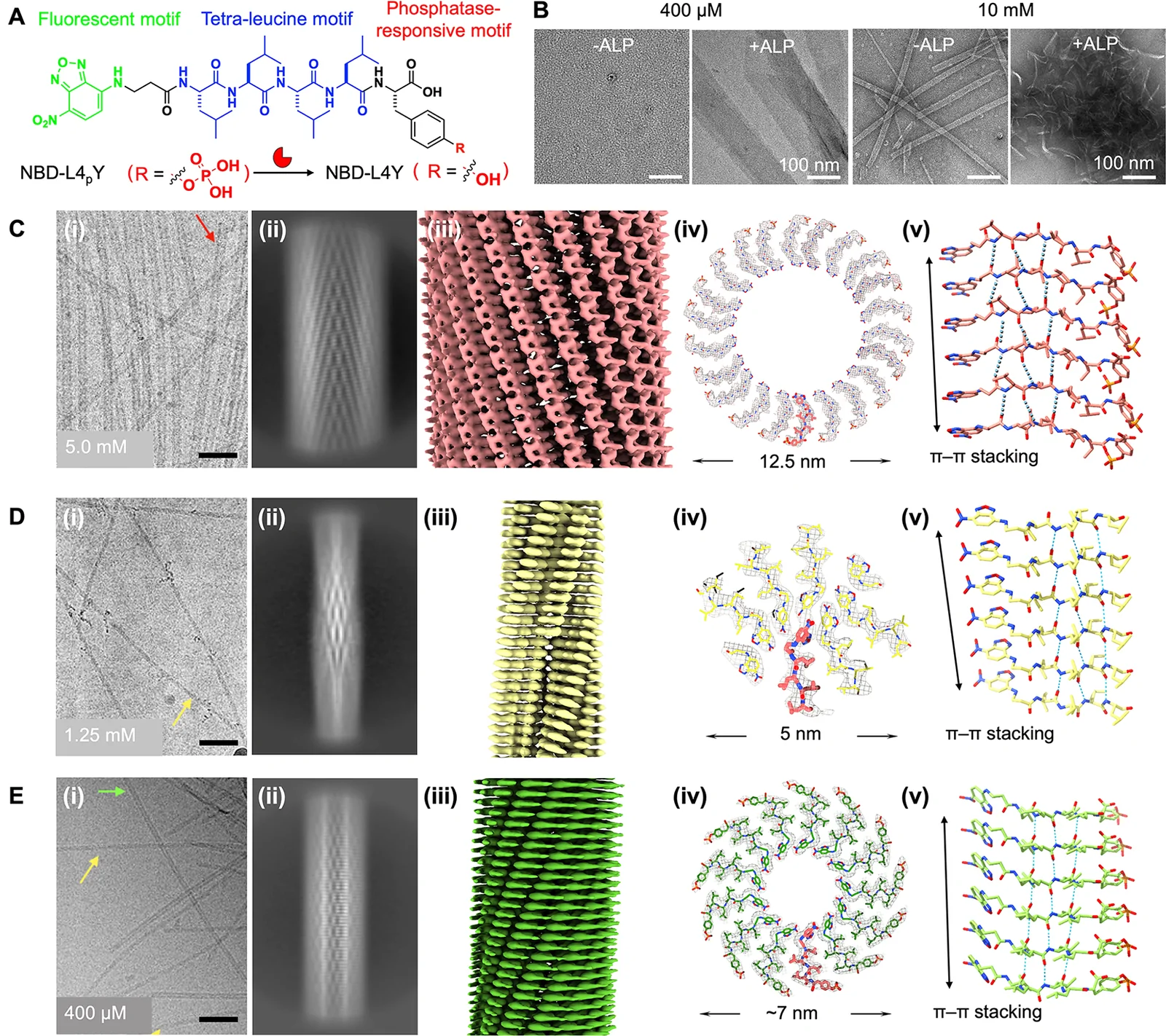
Assembly history encodes a polymorphic peptide landscape in cell-free conditions. (A) Molecular design of NBD-L4_p_Y and schematic of its enzymatic dephosphorylation yielding NBD-L4Y. (B) Transmission electron microscopy images of NBD-L4_p_Y (400 μM or 10 mM) incubated for 24 h in the absence or presence of ALP (1 U/mL). Cryo-EM structural determination of NBD-L4_p_Y at (C) 5.0 mM, (D) 1.25 mM, and (E) 400 μM. From left to right: (i) Representative cryo-EM micrographs, (ii) 2D class averages, (iii) 3D helical reconstructions, (iv) fitted atomic models shown in cross section, and (v) arrays of NBD-L4_p_Y molecules along the filament axis. Scale bars, 50 nm. Arrows in (i) indicate distinct nanofiber morphologies; arrow colors match the backgrounds in (C–E). One peptide subunit is highlighted in red in each structure in (iv), and dashed lines in (v) indicate hydrogen bonds.

We next asked whether these product morphologies reflect the structural state of the phosphorylated precursor before enzymatic conversion. Nanofilaments prepared at 5.0 mM were diluted to 1.25 mM and 400 μM immediately before vitrification, allowing us to test how modest concentration shifts redirect the assembly pathway. At 5.0 mM, cryo-EM showed predominantly hollow nanotubes with 11–14 nm diameters (Figure 1C­(i)). Classification and power-spectrum analysis identified a dominant ∼12.5 nm nanotube population (Figures 1C­(ii) and S4A).^40,41^ The reconstruction places NBD groups toward the lumen and phosphotyrosines on the exterior (Figure 1C­(iii,iv)). Within this architecture, leucine-rich lateral contacts form a predominantly hydrophobic interface that can accommodate variations in molecular packing, providing a structural basis for the observed diameter plasticity.^42,43^ In contrast, axial assembly is reinforced by parallel β-sheet hydrogen bonding, giving rise to the cross-β architecture characteristic of amyloid-like assemblies (Figure 1C­(v)).^44,45^

Dilution to 1.25 mM produced a highly homogeneous ∼5 nm nanofiber with C2 symmetry (Figure 1D­(i–iii)). This filament retains similar assembly profiles: an NBD-rich core, surface-exposed phosphotyrosines, hydrophobic lateral packing, and axial cross-β stabilization, but with a tighter cross-section and partial peripheral density consistent with increased flexibility (Figure 1D­(iv,v)). The rapid shift from wide nanotubes to narrow fibers shows that the phosphorylated precursor remains structurally plastic under these conditions, with accessible nuclei controlled by concentration.

Further dilution to 400 μM yielded a mixed population in which ∼5 nm nanofibers persisted, but distinct ∼7 nm nanotubes emerged (Figure 1E­(i–ii)). The 7 nm nanotubes display C13 symmetry, placing NBD groups within the lumen and phosphotyrosines on the surface (Figure 1E­(iii,iv)) and are stabilized by a hydrophobic lateral interface together with axial cross-β contacts (Figure 1E­(v)). Thus, a small concentration range gives access to multiple well-ordered helical states rather than a single assembly end point.

Viewed together, the three ordered structures reveal a modular basis for NBD-L4_p_Y polymorphism. Axial cross-β hydrogen bonding and the inward-facing NBD/outward-facing phosphotyrosine topology are conserved, whereas lateral packing varies to generate fibers and nanotubes with distinct diameters and symmetries. The conserved axial interface therefore provides a common polymerization scaffold, while the more adaptable lateral interface permits changes in global morphology. These structures define a polymorphic landscape in which multiple ordered states are accessible rather than a single concentration-defined assembly end point.

The preparation pathway was critical for accessing this landscape. Samples prepared directly at 400 μM and incubated at 37 °C showed no detectable filamentous assemblies (Figure 1B). In contrast, samples diluted from 5.0 mM to 1.25 mM or 400 μM immediately before vitrification contained abundant, well-ordered filaments. Thus, samples with the same final concentration can display markedly different assembly states depending on their kinetic history. Rapid dilution followed by immediate vitrification therefore captures transient structures populated along the dilution pathway. These findings motivate direct *in situ* analysis to determine whether the bioactive pericellular environment favors one of the cell-free polymorphs, maintains a heterogeneous ensemble, or generates a distinct supramolecular architecture with different molecular packing.

### NBD-L4_p_Y Forms Homogeneous Pericellular Nanotubes *in Situ*

Given the polymorphic landscape defined *in vitro* and the absence of detectable nanotubes in the equilibrated working solution, the key question is which architecture becomes enriched at the earliest peptide-cell interface. We therefore treated Saos-2 cells with NBD-L4_p_Y at the validated osteosarcoma-inhibitory concentration^34^ and vitrified after 5 min, capturing early assemblies generated at the pericellular niche before extensive intracellular remodeling (Figure 2A).

**Figure 2:**
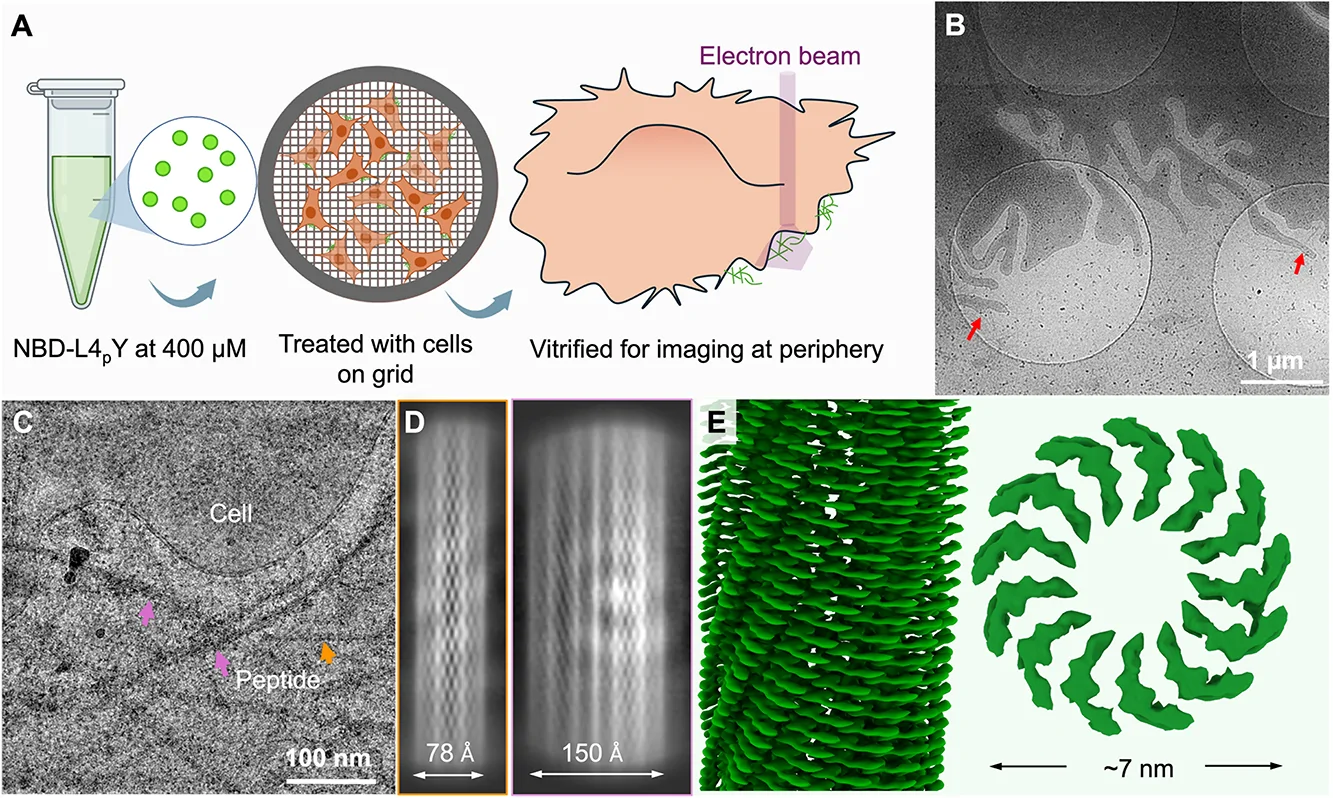
The pericellular niche selects a predominant ∼7 nm peptide nanotube *in situ*. (A) Schematic illustration of *in situ* cryo-EM imaging of peptide nanofilaments generated at the cell periphery. (B) A low-magnification cryo-EM image of the cell periphery in the treatment of NBD-L4_p_Y. Red arrows indicate peptide filaments, which were selected as target regions for data collection. (C) A cryo-EM micrograph and (D) 2D class average of peptide filaments and bundles at the cell periphery. The orange arrow indicates peptide filament. The magenta arrows indicate filament bundles. (E) Side and cross-section views of the 3D reconstruction of the *in situ* filaments.

We targeted filament-rich pericellular regions, particularly those near membrane invaginations, for data collection (Figure 2B). Representative micrographs revealed abundant nanofilaments adjacent to the plasma membrane (Figure 2C). 2D classification yielded a dominant filament population with an apparent diameter of ∼8 nm, together with minor classes consistent with closely packed bundles of ∼15 nm (Figure 2D). The bundled species were absent from the cell-free precursor samples and are consistent with local dephosphorylation, which reduces interfilament electrostatic repulsion and promotes lateral packing. The *in-situ* class averages and averaged power spectra closely matched those of the ∼7 nm nanotube captured after rapid dilution *in vitro*. Together with their shared C13 symmetry and closely similar helical parameters, these observations support assignment of the two assemblies to the same C13 packing architecture (Figure S5 and Table S1).

Helical parameters were indexed from the averaged power spectrum and refined iteratively, yielding a cross-β helical reconstruction with C13 symmetry. The refined parameters (rise 4.65 Å; twist 4.17°) differ slightly from those of the *in vitro* nanotube (rise 4.80 Å; twist 4.05°) (Figure 2E). These data identify the ∼7 nm C13 nanotube as the predominant cell-selected pericellular structure of NBD-L4_p_Y. Notably, this pericellular nanotube could contain partially dephosphorylated species, as the phosphorylated peptide and its dephosphorylated product may share the same assembly backbone.^46^ However, their co-assembly prevents an unambiguous assignment, as the phosphate signal could be averaged out during reconstruction. The pericellular assembly profile differs from both the mixed 5–7 nm ensemble generated by *in vitro* dilution and the equilibrated 400 μM bulk sample, in which no filamentous assemblies were detected. The pericellular environment therefore does not simply reproduce the cell-free landscape. Instead, it preferentially enriches an accessible C13 architecture at the earliest peptide–cell interface. The bundled classes observed *in situ* may represent a further reaction-associated state promoted by cellular dephosphorylation and local enrichment.

### Pericellular Nanotubes Associate with Plasma-Membrane Perturbation during Early Exposure

Having identified the ∼7 nm nanotube as the cell-selected pericellular structure, we next asked whether this structure accounts for the earliest membrane-level function of NBD-L4_p_Y. Single-particle cryo-EM provides the filament structure, but not the three-dimensional membrane context, and individual early filaments are below the practical detection limit of fluorescence imaging. We therefore used cryo-ET to visualize pericellular assemblies and their spatial relationship to the plasma membrane.

To capture early interactions before extensive filament accumulation, Saos-2 cells on EM grids were exposed to NBD-L4_p_Y for 2 min and then vitrified. Cryo-EM images of the cell periphery revealed filopodia, plasma-membrane invaginations, and extracellular vesicles, with filamentous assemblies located near the engulfing membrane (Figure 3A,B and Video S1). Segmentations placed filaments above and below membrane invagination sites (Figure 3C,D and Video S2). At this stage, cellular components such as ribosomes, microtubules, coated vesicles, actin filaments, and mitochondria appeared intact (Figures 3A–D and S6), indicating that peptide-membrane contacts precede global ultrastructural collapse.

**Figure 3:**
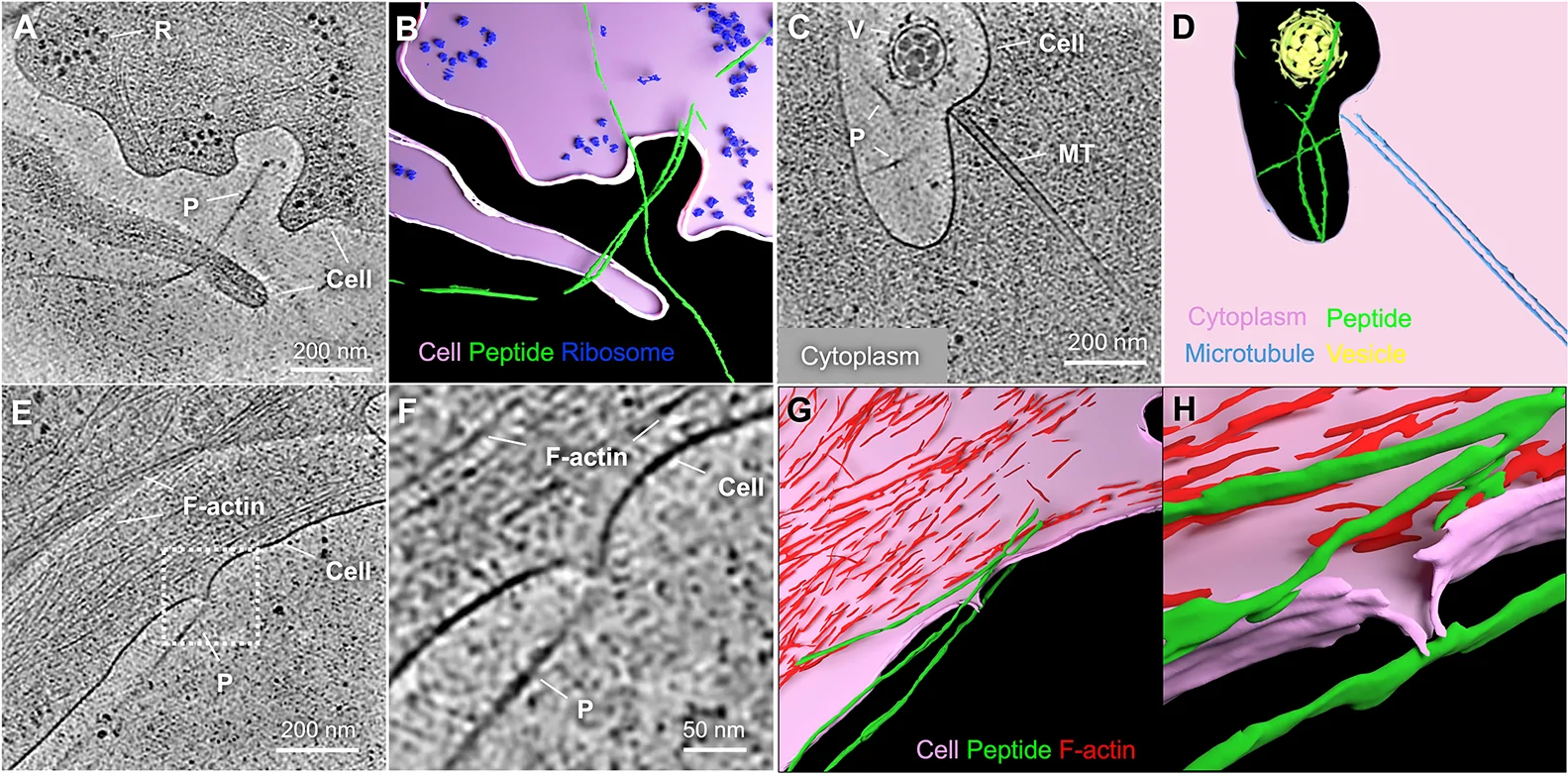
Cell-selected pericellular nanotubes engage and perturb the plasma membrane. (A,C) Reconstructed tomograms and (B,D) corresponding segmentations of the Saos-2 cell periphery after 2 min of NBD-L4_p_Y treatment. (E) Tomogram slice showing a peptide filament at a plasma-membrane contact site. (F) Magnified view of the boxed region in (E). (G) Segmentation and (H) magnified 3D rendering of the peptide-membrane contact site shown in (E,F). Panel labels: P, peptide; R, ribosome; MT, microtubule; V, vesicle; F-actin, filamentous actin.

Notably, we captured a plasma-membrane protrusion in direct contact with filamentous assemblies (Figure 3E–H). Sequential *z*-slices showed a focal reduction in membrane electron density at the peptide-contact site (Video S3), consistent with localized nanoscale membrane perturbation. Together with the subsequent dye-entry experiments, these observations reveal a spatial and temporal association between pericellular filament accumulation, focal membrane perturbation, and peptide entry.

### Peptide Entry is Followed by Redistribution to Intracellular Compartments

To follow the subsequent cellular entry and intracellular itinerary of NBD-L4_p_Y at later time points, we monitored the peptide uptake by confocal laser scanning microscopy (CLSM). At 400 μM, NBD-L4_p_Y rapidly accumulated near the cortical regions within 5 min, followed by redistribution to the nuclear envelope by 25 min, and pronounced nuclear localization by 30 min (Figure 4A). The imaging was performed without washing, and the peptide remained in the medium throughout acquisition, accounting for the diffuse extracellular fluorescence observed in these images. By contrast, at 5 mM, the peptide showed little cellular entry, with only a few cells displaying background fluorescence after 30 min (Figure 4B). This concentration dependence is functionally important: higher peptide concentrations do not necessarily enhance uptake, consistent with the idea that the bioactive state is a specific cell-selected assembly rather than bulk peptide abundance.

**Figure 4:**
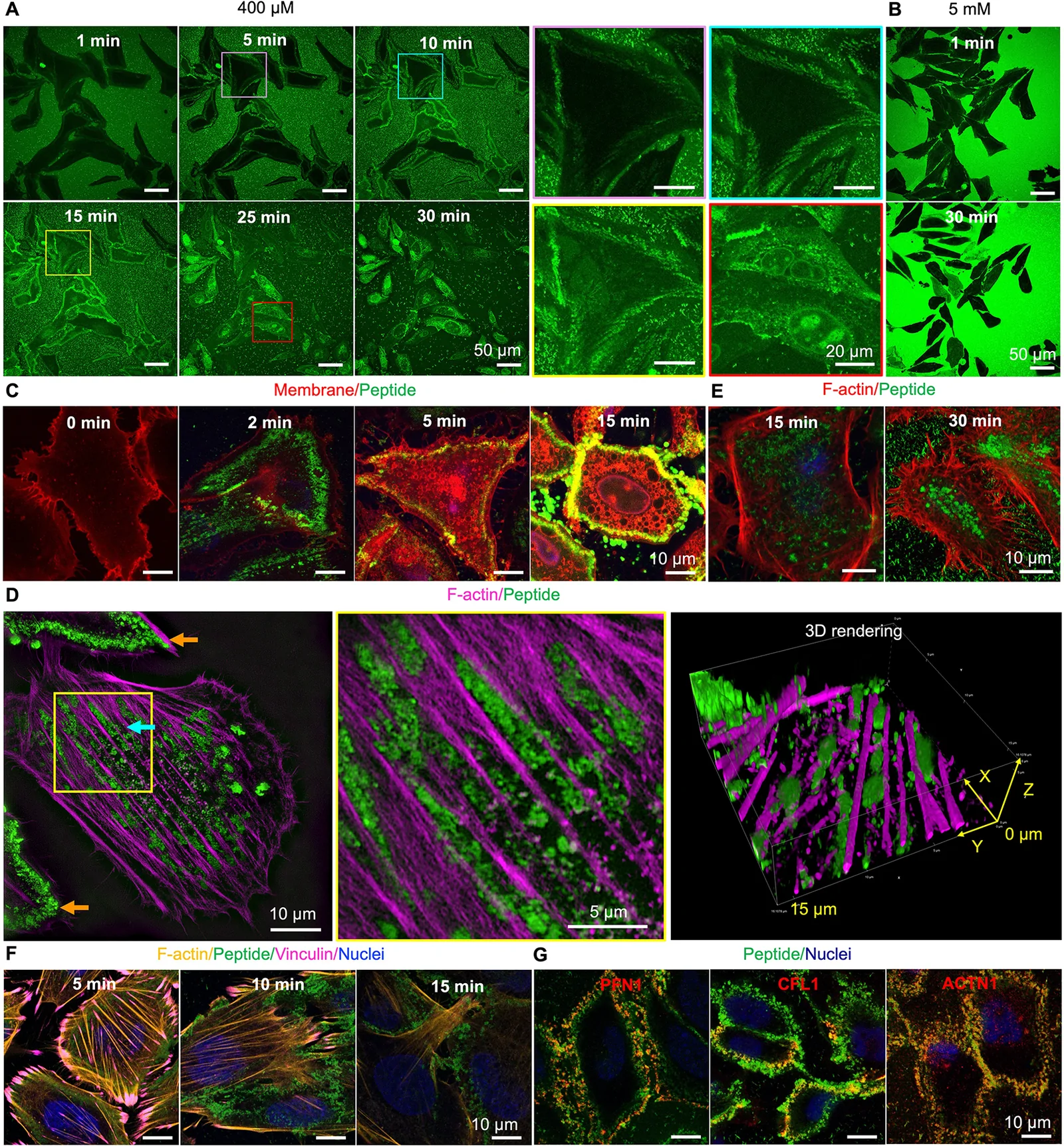
Cellular entry of NBD-L4_p_Y drives subcellular redistribution and cytoskeletal perturbation. Confocal images of Saos-2 cells treated with NBD-L4_p_Y at (A) 400 μM or (B) 5 mM over the course of 30 min. (C) Confocal images of Saos-2 cells prestained with a plasma-membrane dye and then treated with NBD-L4_p_Y for 2 to 15 min (D) SIM images of cells prestained with Actin Tracker and then treated with NBD-L4_p_Y for 5 min. Orange arrows indicate the cortical-rim distribution of peptide assemblies; the cyan arrow indicates dispersed intracellular assemblies. (E) Confocal images of cells treated with NBD-L4_p_Y for 15 or 30 min, followed by staining of F-actin and nuclei. (F) Confocal images of cells treated with NBD-L4_p_Y for 5 to 15 min, followed by staining of vinculin, F-actin, and nuclei. (G) Immunofluorescence staining of PFN1, CFL1, and ACTN1 in cells treated with NBD-L4_p_Y for 15 min [NBD-L4pY] = 400 μM.

Membrane morphology and dye-entry assays further showed permeability changes during this interval. Peptide exposure induced prominent blebbing (Figure S7). In cells prestained with a membrane-impermeable dye, peptide signal appeared within 2 min while membrane fluorescence decreased (Figure 4C and Video S4). Between 5 and 15 min, dye entered intracellular compartments and stained cytoplasmic vacuoles (Figure 4C).

Organelle probes revealed that entry was followed by broader intracellular stress. Endoplasmic reticulum (ER) morphology shifted toward vacuoles and puncta (Figure S8A), mitochondria lost elongated rod-like morphology and showed reduced signal intensity (Figure S8B), and lysosomes became less motile while retaining a punctate appearance (Figure S8C and Video S5). Reduced lysosomal motility suggested that peptide accumulation affects cytoskeleton-dependent trafficking, motivating us to analyze the actin and microtubule networks.

### Cortical Peptide Assemblies Disrupt Actin Organization and Redistribute Actin-Binding Proteins

Live-cell imaging revealed an intracellular peptide rim at the plasma membrane and, in some cells, a radiating fibrillar pattern (Figure S9). Structured illumination microscopy (SIM) imaging after 5 min resolved two related distributions: a continuous cortical peptide rim and dispersed intracellular peptide assemblies that preferentially occupied spaces between actin stress fibers (Figure 4D and Video S6). 3D rendering confirmed a complementary spatial relationship between peptide assemblies and F-actin. This spatial organization preceded rapid cytoskeletal failure: cortical actin disassembled by 15 min, cells lost polarity and formed extensive filopodia by 30 min (Figure 4E), and stress fibers were disrupted by 1 h (Figure S10). Consistent with loss of actin-based adhesion, vinculin patches at F-actin termini decreased between 5 and 10 min and were largely absent by 15 min (Figure 4F).

Microtubules were also affected during the same window. NBD-L4_p_Y treatment produced collapsed, fragmented, and largely immobile microtubules (Video S7), consistent with reduced lysosome motility (Figure S8C and Video S5). Bright-field images showed that cells remained extended during treatment (Figure S11), supporting that cytoskeletal disruption was not simply a secondary consequence of cell shrinkage.

To identify molecular links between peptide assemblies and actin remodeling, we analyzed actin-associated proteins and the relevant phosphatase context, which includes PFN1, cofilin-1 (CFL1), α-actinin-1 (ACTN1), and α-actinin-4 (ACTN4).^47^ Tissue-nonspecific alkaline phosphatase (ALPL), overexpressed in Saos-2 cells,^48^ was examined because it catalyzes the dephosphorylation of NBD-L4_p_Y, and inhibition of this activity abolishes intracellular accumulation and cytotoxicity.^35^ PFN1, CFL1, and ACTN1 showed variable colocalization with peptide assemblies, whereas ACTN4 showed little overlap (Figures 4G and S12). PFN1 was initially dispersed throughout untreated cells (Figure S13A). Within 5 min, PFN1 became depleted from some cytoplasmic regions and enriched at peptide-rich cortical regions (Figure S13B,C). By 15 min, PFN1 was largely restricted to the basal plane, where it extensively overlapped with peptide assemblies (Figure S13D). Signals for phosphorylated profilin-1 (*p*-PFN1) and phosphorylated cofilin-1 (*p*-CFL1) decreased after peptide treatment (Figure S14). Because membrane permeability is already compromised during peptide entry (Figure 4C), ecto-phosphatases such as ALPL may access intracellular substrates and contribute to altered phosphorylation. Partial ALPL colocalization with peptide assemblies (Figure S15) further links enzymatic conversion, peptide accumulation, and remodeling of the cortical actin-regulatory environment.

### Intracellular Remodeling Generates Dense Peptide Assemblies that Disrupt F-Actin and Organelles

We next asked whether peptide assemblies retain the pericellular nanotube morphology after entry or instead remodel into a distinct intracellular functional state. Fluorescence microscopy established the timing and subcellular distribution of peptide accumulation, but not the nanoscale morphology or physical interfaces of the intracellular assemblies. We therefore performed cryo-ET on cryo-FIB-milled lamellae to visualize NBD-L4_p_Y inside cells *in situ*.

Cells were incubated with peptide for 15 min, when intracellular accumulation and actin-associated peptide patterns were already apparent (Figure 4D). The actin-rich cortex was thinned by cryo-FIB milling to lamellae below 200 nm (Figure 5A,B) and scanning electron microscopy (SEM) confirmed that the lamellae contained intracellular compartments and organelles suitable for cryo-ET analysis (Figure 5C).

**Figure 5:**
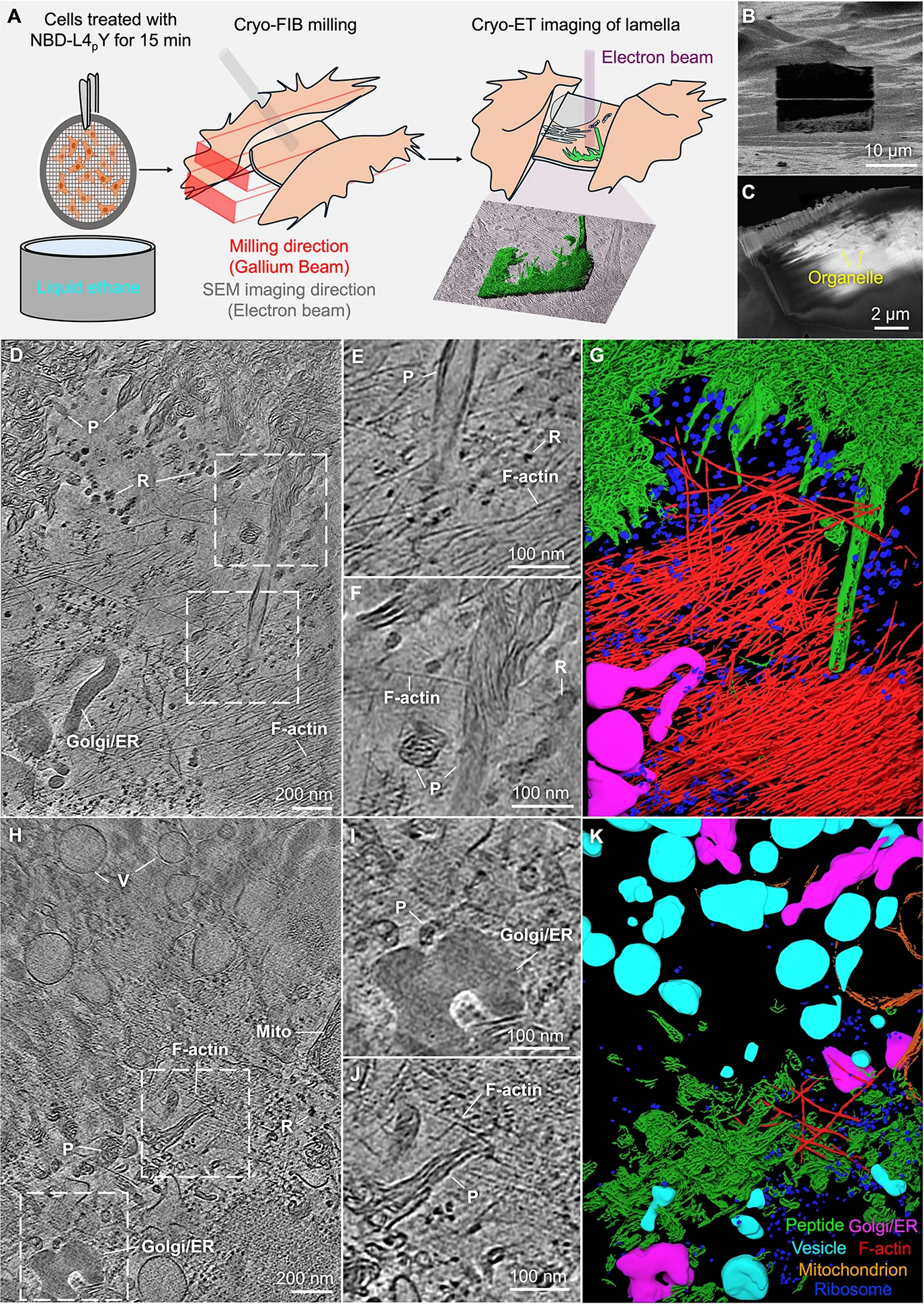
Intracellular peptide remodeling generates dense assemblies that interface with F-actin and membranous organelles. (A) Workflow for preparing Saos-2 cells on gold EM grids for cryo-FIB milling and cryo-ET. (B) Representative SEM image of a milled cellular region. (C) SEM image of a finished lamella. (D,H) Reconstructed tomograms from lamellae of cells treated with NBD-L4_p_Y for 15 min. (E,F,I,J) Magnified views of the boxed regions in (D,H). (G,K) Corresponding segmentations showing interfaces between peptide assemblies and intracellular structures. Panel labels: P, peptide; R, ribosome; V, vesicle; F-actin, filamentous actin; Golgi/ER, Golgi apparatus/endoplasmic reticulum; Mito, mitochondrion.

Reconstructed tomograms showed densely packed, entangled peptide assemblies localized near F-actin, along with organelles, such as Golgi/ER, and ribosomes (Figure 5D–F and Video S8). Segmentations showed rod-like peptide densities protruding into the F-actin network, where they were positioned to obstruct or reorganize actin filaments (Figures 5G and S16A). These intracellular assemblies were morphologically distinct from the pericellular nanotube. Moreover, their morphology differed from the 400 μM particle-to-ribbon transition observed *in vitro* and more closely resembled high-concentration mixtures of ribbons and curvatures (Figure S3). The clear ultrastructural distinction between the well-ordered pericellular nanotubes and the heterogeneous, curved intracellular assemblies supports substantial supramolecular remodeling associated with cell-mediated enzymatic conversion. However, their heterogeneous organization, crowded packing, and limited sampling in cryo-FIB lamellae currently impede high-resolution reconstruction, which will require larger targeted datasets and a sufficiently ordered structural subclass. Although the structural transition from pericellular nanotubes to intracellular assemblies was not directly resolved *in situ*, the observed supramolecular remodeling may involve multiple processes, including disassembly and reassembly, enzymatic conversion, local peptide enrichment, and interactions with intracellular proteins.

Additional lamellae linked this remodeled state to organelle damage. Vesicle accumulation, ER/Golgi-associated peptide contacts, and mitochondria with discontinuous membrane bilayers were observed in peptide-treated cells (Figure 5H–J and Video S9). Segmentations also showed peptide assemblies in contact with Golgi/ER membranes and fragmented actin filaments embedded within the assemblies (Figures 5K and S16B). These observations define the second stage of the mechanism: after membrane entry, NBD-L4_p_Y remodels into dense intracellular assemblies that physically interface with F-actin and membranous organelles, placing the material in position to disrupt cytoskeletal organization and organelle integrity.

At 30 min, cryo-ET further revealed intracellular peptide assemblies near the nuclear envelope, which exhibited abnormal dilation consistent with cellular stress (Figure S17).^49^ This spatial organization provides an ultrastructural context for the pronounced nuclear accumulation observed by 30 min.

Moreover, tomograms of control Saos-2 cells showed no evidence of the dense, entangled peptide assemblies or filamentous peptide nanotubes observed in peptide-treated cells (Figure S18). Classic cellular structures, including plasma membrane, F-actin, vesicles, ER, and mitochondria, remained readily distinguishable. These results further support the assignment of the structures observed after NBD-L4_p_Y treatment to peptide-derived assemblies.

### Biochemical Assays Link Peptide Assemblies to PFN1 Redistribution and Impaired Actin Polymerization

The imaging results suggested a potential link between intracellular peptide deposits and altered actin regulation. We examined this association by combining biochemical fractionation, Western blotting (WB), LC–MS, and cell-free assays of peptide-actin interactions. Saos-2 cells were incubated with NBD-L4_p_Y for varying durations before being washed, lysed, and centrifuged to separate supernatant and pellet fractions for electrophoresis (Figure 6A). The lysate pellets increased in volume over time (Figure 6B), consistent with progressive cellular deposition of peptide assemblies from 5 min to 1 h.

**Figure 6:**
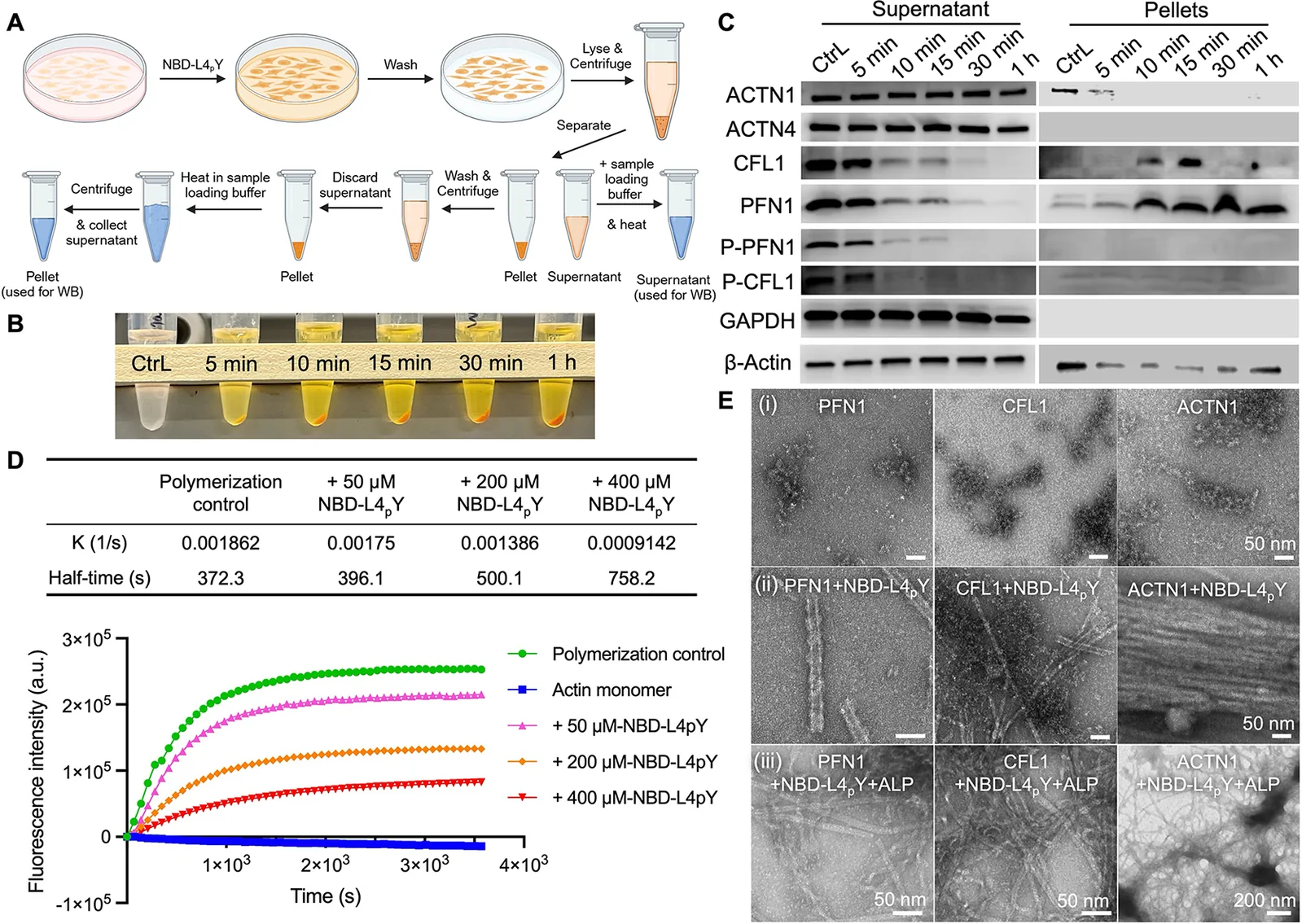
Biochemical validation links peptide assemblies to PFN1 redistribution and impaired actin polymerization. (A) Workflow for preparing supernatant and pellet fractions from Saos-2 cells for WB analysis. (B) Optical image of cells treated with peptide for the indicated durations, followed by lysis and centrifugation. (C) WB analysis of supernatant and pellet fractions from (B) across the time course. (D) Actin polymerization assay showing conversion of monomers in the presence of peptide (50–400 μM). Data were fitted with a one-phase association model to calculate rate constant (K) and half-time. (E) TEM images of actin-binding proteins (i) PFN1, CFL1, and ACTN1 alone (0.4 mg/mL), (ii) after coincubation with peptide (400 μM); and (iii) after coincubation with peptide (400 μM) and ALP (1 U/mL).

LC–MS analysis of pellet fractions confirmed the identity of these deposits. From 15 min to 1 h, the dominant species was the dephosphorylated product NBD-L4Y, with residual NBD-L4_p_Y also detected (Figures S19–S21). Thus, intracellular deposits arise predominantly from ALPL-driven conversion rather than simple accumulation of the phosphorylated precursor.

WB analysis then connected these deposits to actin regulators. CFL1 and PFN1 decreased rapidly in the soluble fraction during peptide exposure, together with reduced *p*-CFL1 and *p*-PFN1 signals, whereas ACTN1 and ACTN4 remained comparatively stable (Figure 6C). PFN1 showed the strongest enrichment in the pellet fraction, indicating that it is the protein most prominently redistributed from the soluble fraction into peptide-rich deposits. In contrast, ACTN1, ACTN4, *p*-PFN1, and *p*-CFL1 showed weak pellet signals. Low GAPDH and β-actin signals in the pellets confirmed effective fractionation and supported the specificity of PFN1 enrichment.

To evaluate whether the peptide influences actin dynamics directly, we conducted cell-free actin polymerization assays^50,51^ by monitoring the fluorescence enhancement of pyrene-labeled globular actin (G-actin). In polymerization buffer, G-actin produced the expected increase in pyrene fluorescence. NBD-L4_p_Y delayed the pyrene fluorescence increase in a dose-dependent manner, as reflected by a decreased rate constant and extended half-time (Figure 6D). Because ALP interferes with the ATP-dependent actin polymerization assay and NBD-L4Y forms heterogeneous assemblies that complicate quantitative fluorescence measurements, the pyrene assay was performed with NBD-L4_p_Y under defined cell-free conditions. Rhodamine-actin imaging gave the same functional conclusion: NBD-L4_p_Y suppressed filament formation, whereas ALP treatment promoted actin clustering (Figure S22A). TEM further showed fragmented actin filaments associated with peptide nanotubes or twisted peptide ribbons after ALP treatment (Figure S22B). These experiments establish that the peptide is able to impair actin polymerization and to engage actin filaments outside the cellular context.

TEM co-assembly experiments explained why protein identity matters. PFN1, CFL1, and ACTN1 alone appeared amorphous (Figure 6E­(i)), but each redirected NBD-L4_p_Y assembly differently. PFN1 appeared as particles decorating the surface of NBD-L4_p_Y nanotubes, CFL1 formed aggregates independent of the fibrils, and ACTN1 promoted the formation of closely packed peptide bundles with minimal dispersed aggregates (Figure 6E­(ii)). Enzymatic dephosphorylation of NBD-L4_p_Y by ALP further remodeled these assemblies, producing curly fibrils alongside tubular structures for PFN1 and CFL1 and larger, densely packed bundles for ACTN1 (Figure 6E­(iii)). These co-assembly morphologies differ from the particle-to-ribbon transition observed for NBD-L4_p_Y alone, demonstrating that actin-binding proteins can reshape the peptide assembly profile. The biochemical and TEM data therefore support a model in which peptide assemblies are associated with PFN1 redistribution into peptide-rich deposits and impaired actin polymerization, while protein-dependent co-assembly may further contribute to cytoskeletal disruption.

## Conclusion

By resolving NBD-L4_p_Y from the molecular to the cellular scale, this study links biological activity to a context-selected supramolecular state. Cell-free cryo-EM defined a concentration- and history-dependent polymorphic landscape, whereas *in situ* single-particle cryo-EM identified a predominant ∼7 nm C13 nanotube at the pericellular interface at 3.4 Å resolution. Cryo-ET localized this nanotube to membrane-contact and invagination sites associated with focal membrane perturbation and an early route for entry. Following entry, cryo-ET of cryo-FIB-milled lamellae revealed a transition to dense, morphologically distinct intracellular assemblies that interface with F-actin and membranous organelles. Biochemical fractionation and reconstitution further connected these deposits to PFN1 redistribution and impaired actin polymerization. Together, these results support a two-stage trajectory in which the cellular environment first selects a membrane-active polymorph from an accessible cell-free landscape and then remodels the peptide into intracellular assemblies that disrupt actin regulation.

The central advance is therefore not simply the visualization of enzyme-triggered assembly or a terminal cell-death phenotype, but the direct structural assignment of a cell-selected synthetic peptide polymorph and the mapping of its subsequent remodeling across biological compartments. The integrated workflow resolves information unavailable to any single method:^36^ cell-free cryo-EM defines accessible states; *in situ* cryo-EM assigns the predominant pericellular architecture; cryo-ET reveals the pericellular and intracellular assemblies within membrane, cytoskeletal, and organelle contexts; cryo-FIB milling provides access to the thicker cellular interior; and live-cell imaging and biochemical assays connect structural transitions to cellular consequences.

More broadly, this study illustrates a general principle for responsive soft nanomaterials generated through reaction-diffusion processes.^38^ Biological function can arise not only from the chemical trigger itself, but from how that trigger is translated into distinct supramolecular states at specific cellular locations and within narrow temporal windows. This differs from paradigms where enzyme activation primarily serves as an on–off switch that generates a “effector” morphology whose activity is largely defined by one terminal failure mode, such as direct membrane disruption.^33^ Instead, NBD-L4_p_Y highlights a context-selected, multi-stage trajectory, indicating that enzymatic reactions confer functions to dynamic structures. The ability to resolve transient, context-dependent supramolecular states across space and time opens new opportunities for designing nanostructures that exploit biological triggers, structural plasticity, and protein interactions for therapeutic applications. Recent advances in *in situ* single-particle cryo-EM have enabled near-atomic structural determination of proteins directly in native environments, including the red algal megacomplex,^52^ mammalian respiratory supercomplexes,^53^ the bacterial secretion system,^54^ and beta-amyloids.^55^ In parallel, studies of β-amyloid assemblies have underscored the importance of model systems that faithfully recapitulate the structures selected *in situ* for understanding molecular mechanisms and informing diagnostics and therapeutics.^56^ This work advances that principle to synthetic supramolecular materials and highlights the importance of resolving context-selected structures directly in cellular environments. By integrating structural precision with cellular function, multiscale imaging moves beyond descriptive visualization to reveal principles that can guide the rational development of supramolecular therapeutics and biomaterials.

## Supplementary Material





















## Notes

Experimental procedures; peptide characterization; additional microscopy, biochemical, and structural data; and cryo-EM processing and refinement statistics (PDF); Supporting Information Videos S1−S9 showing cryo-ET and live-cell imaging of peptide–membrane interactions, cellular entry, cytoskeletal perturbation, and intracellular peptide remodeling:
Supporting Information Video S1 (AVI)
Supporting Information Video S2 (AVI)
Supporting Information Video S3 (AVI)
Supporting Information Video S4 (MP4)
Supporting Information Video S5 (MP4)
Supporting Information Video S6 (MP4)
Supporting Information Video S7 (MP4)
Supporting Information Video S8 (MP4)
Supporting Information Video S9 (MP4)

## Abbreviations

ACTN1: α-actinin-1
ACTN4: α-actinin-4
ALP: alkaline phosphatase
ALPL: tissue-nonspecific alkaline phosphatase
CFL1: cofilin-1
CLSM: confocal laser scanning microscopy
cryo-EM: cryo-electron microscopy
cryo-ET: cryo-electron tomography
cryo-FIB: cryogenic focused ion beam
EISA: enzyme-instructed self-assembly
EM: electron microscopy
ER: endoplasmic reticulum
F-actin: filamentous actin
G-actin: globular actin
HPLC: high-performance liquid chromatography
L4: tetra-leucine
LC–MS: liquid chromatography–mass spectrometry
NBD: 7-nitrobenzofurazan
*p*-CFL1: phosphorylated cofilin-1
*p*-PFN1: phosphorylated profilin-1
PFN1: profilin-1
_p_Y: phosphotyrosine
SEM: scanning electron microscopy
SIM: structured illumination microscopy
TEM: transmission electron microscopy
WB: Western blotting
