# Supplementary material for: In Situ Multiscale Imaging Reveals Cellular Selection and Remodeling of Peptide Assemblies from Membrane Perturbation to Actin Dysregulation
Source: J Am Chem Soc. 2026 Sep 10;148(37):40407–18. doi: 10.1021/jacs.6c14737 (PMC13614308; doi:10.1021/jacs.6c14737)
Supplement: Supplementary file 1 [file ja6c14737_si_010.pdf]

Supporting Information for

In situ multiscale imaging reveals cellular selection and remodeling of peptide assemblies from membrane perturbation to actin dysregulation

Jiaqi Guo[1,3,4,*], Ayisha Zia[2], Wangbiao Guo[3,4], Shoichi Tachiyama[3,4], Kangqiang Qiu[6], Jiaqi Wang[3,4], Jack Botting[3,4], Zhiyu Liu[1], Yuchen Qiao[1], Jiajie Diao[6], Jun Liu[3,4], Fengbin Wang[2,5,*], Bing Xu[1,*]

[1]Department of Chemistry, Brandeis University, 415 South St., Waltham, MA, 02453, USA

[2]Department of Biochemistry and Molecular Genetics, University of Alabama at Birmingham, Birmingham, AL, 35294, USA

[3]Department of Microbial Pathogenesis, Yale School of Medicine, New Haven, CT 06536, USA

[4]Microbial Sciences Institute, Yale University, West Haven, CT 06516, USA

[5]O'Neal Comprehensive Cancer Center University of Alabama at Birmingham, Birmingham, AL, 35294, USA

[6]Department of Cancer Biology, Center for Chemical Imaging in Biomedicine, Advanced Cell Analysis Service Center, University of Cincinnati College of Medicine, Cincinnati OH, 45267, USA

Methods

Materials

2-Chlorotrityl chloride resin (1.02 mmol/g), Fmoc-protected amino acids, and 2-naphthaleneacetic acid were from GL Biochem (Shanghai, China). O-(Benzotriazol-1-yl)-N,N,N′,N′-tetramethyluronium hexafluorophosphate (HBTU) was from Chem-Impex; N,N-diisopropylethylamine (DIPEA) from TCI America; and 4-chloro-7-nitrobenzofurazan from Alfa Aesar. Alkaline phosphatase (Lot# 1700ACX2) was from Biomatik. Hoechst 33342 was from Molecular Probes. Tris-glycine transfer buffer (25×, catalog #LC3675) was from Invitrogen.

CellMask™ Deep Red Actin Tracking Stain (catalog #A57245), CellMask™ Plasma Membrane Stain (catalog #C10046), α-actinin (ACTN1) monoclonal antibody (OTI7A4, TrueMAB™, catalog #TA500072), α-actinin-4 recombinant rabbit monoclonal antibody (JU20-23, catalog #MA5-32794), phospho-profilin-1 (Ser138) polyclonal antibody (catalog #PA5-143752), GAPDH monoclonal antibody (6C5, catalog #AM4300), SuperBlock™ Blocking Buffer (catalog #37535), ALPL recombinant rabbit monoclonal antibody (7H11L3, catalog #702454), Image-iT™ FX Signal Enhancer (catalog #I36933), Restore™ PLUS Western Blot Stripping Buffer (catalog #46428), and Halt™ protease inhibitor cocktail (100×, catalog #78430) were from Thermo Fisher Scientific.

Actin Cytoskeleton/Focal Adhesion Staining Kit (Millipore Sigma), TWEEN®-20 (catalog #P1379), and Immobilon™-FL PVDF membranes (catalog #IPFL00005) were from Millipore Sigma. Profilin-1 antibody (catalog #3237), cofilin (D3F9) XP® rabbit mAb (catalog #5175), phospho-cofilin (Ser3) (77G2) rabbit mAb (catalog #3313), and RIPA buffer (10×, catalog #9806) were from Cell Signaling Technology. Anti-β-actin antibody (catalog #ab8227), goat anti-rabbit IgG H&L (Alexa Fluor® 647) pre-adsorbed (catalog #ab150083), and goat anti-rabbit IgG H&L (HRP) (catalog #ab6721) were from Abcam.

MiniPROTEAN® TGX™ precast gels, 4× Laemmli sample buffer (catalog #1610747), and 10× Tris/Glycine/SDS electrophoresis buffer were from Bio-Rad. Rabbit skeletal muscle actin (rhodamine, catalog #AR05-A), pyrene-labeled actin (catalog #AP05-A), ATP (100 mM stock, catalog #BSA04-001), 10× actin polymerization buffer (catalog #BSA02-001), 1× general actin buffer (catalog #BSA01-001), human recombinant cofilin-1 protein (catalog #CF01-A), and untagged human recombinant profilin-1 protein (catalog #PR02-A) were from Cytoskeleton.

UltraAuFoil 200-mesh gold grids (R2/2) and Quantifoil 200-mesh Au NH2 3 nm C (R2/1) grids were from Quantifoil. McCoy's 5A medium, fetal bovine serum (FBS), and penicillin–streptomycin (PS) were from Gibco. Human α-actinin-1 recombinant protein (catalog #NBP972501MG) was from Novus Biologicals.

Dimethylformamide (DMF), methylene chloride (DCM), trifluoroacetic acid (TFA), triethylamine, and other reagents and solvents were from Fisher Scientific. All reagents and solvents were used as received without further purification.

Instruments

Crude peptides were purified by reverse-phase HPLC (Agilent 1100 Series) using HPLC-grade water (0.1% TFA) and acetonitrile (0.1% TFA) as eluents. Liquid chromatography-Mass spectrometry (LC-MS) spectra were recorded on a Waters Acquity UPLC coupled to a Waters Micromass Quattro mass spectrometer. Confocal laser scanning microscopy was performed on a Zeiss LSM 880. Structured illumination microscopy (SIM) was performed on a Nikon N-SIM (version AR5.11.00, 64-bit). Cryo-focused ion beam (cryo-FIB) milling was carried out on an Aquilos 2 (Thermo Fisher). Transmission electron microscopy (TEM) was performed on a Morgagni 268 microscope. Cryo-electron microscopy (cryo-EM) grids were prepared with a Leica EM GP2 automated plunger. Cryo-EM and in-cell cryo-electron tomography (cryo-ET) were collected on a 300kV Titan Krios (Thermo Fisher) equipped with a K3 direct electron detector (Gatan), and pericellular cryo-ET was collected on a 200 kV Glacios (Thermo Fisher) also equipped with a K3 detector. Fluorescence intensity measurements were acquired on a BioTek UV/Vis/fluorescence plate reader. Blots were scanned with a LI-COR C-DiGit blot scanner.

Solid-phase peptide synthesis

NBD-L4$_p$Y and its enantiomer were synthesized by standard solid-phase peptide synthesis on 2-chlorotrityl resin.[1, 2] Fmoc-O-phosphotyrosine and NBD-β-alanine were prepared as described previously.[3, 4] Peptides were cleaved from the resin with a cocktail of 95% trifluoroacetic acid, 2.5% triisopropylsilane, and 2.5% water for 1 h, concentrated, precipitated in ice-cold diethyl ether, and centrifuged. The resulting orange solid was collected and purified by high-performance liquid chromatography.

Preparation of rhodamine-actin for TEM and CLSM

Rhodamine–actin was resuspended to 0.4 mg mL^{-1} in General Actin Buffer supplemented with 0.2 mM ATP and 1 mM DTT. Polymerization was initiated by adding one-tenth the volume of Polymerization Buffer and incubating at room temperature for 1 h. The polymerized rhodamine–actin was then diluted to 200 μg mL^{-1} for TEM or 50 μg mL^{-1} for CLSM, supplemented with 400 μM NBD-L4$_p$Y. Samples were incubated with or without ALP for 24 h before TEM or CLSM imaging.

Negatively stained TEM

Copper grids (400-mesh) coated with a continuous carbon film were glow-discharged at −20 mA for 30 s. A 5 μL aliquot of sample solution was applied to each grid and incubated for 1 min before removing the

excess with filter paper. Grids were then washed twice with 40 µL droplets of water and stained with 2% uranyl acetate for 20 s, followed by blotting to remove excess stain. The grids were air-dried and imaged on a Morgagni 268 TEM operated at 80 kV.

Liquid-chromatography mass-spectrometry

Peptide identification by LC–MS

For peptide identification, 1 mg of lyophilized powder was dissolved in a 1:1 (v/v) mixture of acetonitrile and water, filtered through a 0.22 µm PVDF membrane, and injected into the LC–MS.

Enzymatic dephosphorylation assays

To assess enzymatic dephosphorylation, peptide-enzyme mixtures prepared in PBS were quenched with an equal volume of methanol, lyophilized, redissolved in methanol, filtered, and mixed 1:1 with water before reverse-phase HPLC analysis on an Agilent 1100 Series system. Solvent A was water containing 0.1% TFA and solvent B was acetonitrile containing 0.1% TFA; the flow rate was 9.0 mL/min. The gradient program was as follows:

**Time (min)**	**Solvent A (%)**	**Solvent B (%)**
0.0	65	35
18.0	5	95
18.5	0	100
23.5	0	100
24.0	65	35
28.0	65	35

Cell-associated peptide dephosphorylation

For analysis of cell-associated peptide, cells were seeded, treated with peptide for the indicated time points, lysed in lysis buffer, and centrifuged at 15,294 × g for 10 min at 4 °C to separate supernatant and pellet fractions. Pellets were washed with PBS and centrifuged again. The final pellets were dissolved in methanol, filtered, and mixed with an equal volume of water prior to LC–MS injection.

Cell culture

Saos-2 cells (ATCC) were cultured in McCoy's 5A medium supplemented with 15% fetal bovine serum (FBS), 100 U mL^{-1} penicillin, and 100 μg mL^{-1} streptomycin at 37 °C in a humidified incubator with 5% CO_2.

Live-cell confocal laser scanning microscopy

Cells were seeded at 1.5×10^5 cells per 3.5 cm confocal dish and cultured for 24 h to allow attachment. For ER, mitochondrial, and lysosomal staining, cells were incubated with the respective probes at 1:1000 dilution in live-cell imaging solution for 30 min, then washed to remove excess dye. The peptide working solution (500 μL) was prepared by diluting the 10 mM stock in PBS into pre-warmed live-cell imaging solution. Cells were transferred to a humidified live-cell imaging chamber at 37 °C and the medium was replaced with the peptide solution. Fluorescence images from multiple channels were collected every 20 s, 30 s or 1 min for 30 min.

SIM

SIM performed on a Nikon N-SIM system (version AR5.11.00 64-bit, Tokyo, Japan) equipped with an Apochromat 100×/1.49 NA oil-immersion objective and solid-state lasers at 488, 561, and 640 nm (15 mW output at the fiber end). Images (512 × 512 pixels) were collected as Z-stacks with 0.2 μm steps and reconstructed with NIS-Elements AR Analysis (version AR5.11.00 64-bit). The 488 nm laser excited MTG and GFP (emission 500–550 nm), and the 640 nm laser excited MTDR (emission 660–735 nm). Saos-2 cells were seeded on glass-bottom dishes (MatTek P35G-1.5-14-C) for 24 h to allow attachment, pretreated with Deep Red Actin Tracking Stain for 30 min, then exposed to peptide for the designated duration, washed with PBS, fixed in 4% paraformaldehyde, and imaged.

Immunofluorescence staining

Cells were washed twice with PBS, fixed in pre-warmed 4% paraformaldehyde for 15 min, and permeabilized with 0.1% Triton X-100 in PBS for 10 min. Image-iT FX signal enhancer was applied for 30 min to reduce non-specific binding, followed by blocking with blocking buffer for 1 h at room temperature. Cells were incubated with primary antibodies diluted in 1% bovine serum albumin/PBS overnight at 4 °C and then with Alexa Fluor–conjugated secondary antibodies (1:5000) for 1 h at room temperature. Nuclei were stained with 1 μg ml^{-1} Hoechst 33342 in PBS for 15 min. Cells were washed three times with PBS after each step, except after blocking, when primary antibodies were added directly. Primary antibody dilutions: profilin-1 (1:50), phosphor-profilin-1 (1:50), cofilin-1 (1:200), phospho-cofilin (1:50), α-actinin-1 (1:100), α-actinin-4 (1:100), ALPL (1:100), vinculin (1:200), TRITC–phalloidin (1:1000), Deep Red Actin Tracking Stain (1:1000, no secondary antibody).

Preparation of cells on grid

200-mesh UltrAuFoil gold grids (R2/2) were glow-discharged at –20 mA for 40 s twice and equilibrated in phosphate-buffered saline for at least 2 h on a glass coverslip. The buffer was then replaced with culture medium, and the grids were incubated overnight at 37 °C. Saos-2 cells were seeded at 1.5×10^4 cells cm^{-2} onto the grids and allowed to attach overnight. Culture medium was replaced with peptide working solution for the designated incubation time. Grids were washed with 10% glycerol–containing culture medium, blotted, and plunge-frozen using a Leica EM GP2 Automatic Plunge Freezer set to 25 °C and 95% humidity with 15 s back-side blotting. Vitrified grids were clipped into Cryo-FIB autogrids with C-clips (FEI) prior to milling and cryo-ET/EM imaging.

Cryo-FIB of cancer cell

Cryo-FIB milling was performed on an Aquilos 2 focused ion beam/scanning electron microscope (Thermo Fisher Scientific). A low-magnification SEM montage of the entire grid was acquired with MAPS software to select cellular regions for milling. Grids were sputter-coated with metallic platinum at 30 mA for 15 s, followed by a 25 s layer of organometallic platinum, and an additional 15 s of metallic platinum to minimize drift from charging. Rough milling of cellular regions was performed at 0.3 nA to ~1 µm, followed by 50 pA to ~500 nm, with final polishing at 30 pA. The resulting lamellae (<200 nm) were examined by SEM, which revealed well-preserved cellular features including intracellular organelles and plasma membranes, and then stored in liquid nitrogen until use.

Cryo-ET

Lamellae were imaged on a Titan Krios microscope (Thermo Fisher Scientific) equipped with a Gatan K3 direct-detection camera, and cell peripheries were imaged on a Glacios microscope (Thermo Fisher Scientific) with the same detector. Tilt series were collected in SerialEM using FASTtomo scripts[5] with a dose-symmetric scheme from +48° to –48° at 3° steps and a total dose of ~70 $e^-/Å^2$. Data were acquired at 2.148 Å/pixel (Krios) or 2.345 Å/pixel (Glacios) with a nominal defocus of ~–6 µm. Image stacks (~33 images each) were motion-corrected in MotionCor2[6] and assembled into drift-corrected stacks in IMOD.[7] Fiducial-less alignment and tomogram reconstruction were performed in IMOD.[8] Denoising was carried out with IsoNet,[9] segmentation with Dragonfly, and 3D renderings were visualized in Dragonfly.

Cryo-EM single particle analysis

In-vitro peptide nanofilaments

Peptide samples were prepared at three concentrations: 5 mM, 1.25 mM, and 0.4 mM. Dilutions were made via gentle pipetting using the same buffer without additional treatment. Aliquots (4.5 µL) were applied to glow-discharged lacey carbon grids and plunge-frozen using a Leica GP2 plunge freezer. The grids were initially screened using a 200 keV Glacios at the UAB cryo-EM facility, and final datasets were collected on a 300 keV Titan Krios equipped with a Gatan K3 direct electron detector at NCEF. Following data collection, micrographs were manually selected for processing based on the presence of visible peptide fibers and optimal ice condition. For the NBD-L4$_p$Y samples, a total of 1,980 (5 mM), 5,120 (1.25 mM), and 1,643 (0.4 mM) movies were selected for subsequent analysis; detailed parameters, including pixel sizes, are provided in Table S1. Briefly, a total electron dose of ~50 electrons/Å^2 was distributed across 40 fractions. The data processing workflow began with motion correction and CTF estimation within cryoSPARC.[10] Particles were automatically picked using the "Filament Tracer" with a 10-pixel shift. Multiple iterations of 2D classification were performed to exclude "junk" particles, such as those corresponding to carbon edges. Potential helical symmetries were determined from the averaged power spectra of aligned raw particles. Various symmetry parameters were tested through trial-and-error until characteristic peptide features, specifically side-chain densities and the distinct separation of β-sheets, became clearly visible.[11] Final resolutions were assessed using map-to-map and model-to-map Fourier Shell Correlation (FSC). For visualization and atomic model building, maps were further sharpened using a negative B-factor, DeepEMhancer,[12] or EMReady.[13] Detailed refinement and validation statistics are summarized in Table S1.

In-situ peptide nanofilaments

Data acquisition was performed on a Titan Krios transmission electron microscope (Thermo Fisher Scientific) equipped with a Gatan K3 direct electron detector and GIF energy filter operated in super-resolution mode (pixel size 0.534 Å). Micrographs were collected in electron-counting mode using SerialEM for multishot pattern acquisition targeting cell peripheries within ice holes. A total dose of 70 e^-/Å^2 was distributed over 40 frames with defocus values from –1.6 to –2.2 µm. Motion correction and CTF estimation were carried out in cryoSPARC. Particles were auto-picked with the filament tracer (20 Å shift) and subjected to multiple rounds of reference-free two-dimensional classification to remove non-peptide-like particles. The remaining *in situ* peptide nanofilaments formed a homogeneous class with clear helical patterns. All possible helical symmetries were indexed from the averaged power spectrum and iteratively tested in cryoSPARC until recognizable peptide features, including side chains, were resolved. Final map resolutions were estimated by both map–map FSC (statistics summarized in Table S1).

Model building of NBD-L4$_p$Y

The NBD-L4$_p$Y nanotube structures reached resolutions of approximately 3.4 Å (5 mM), 3.5 Å (1.25 mM), and 3.8 Å (0.4 mM), as estimated by map-to-map FSC. Unlike structures containing α-helices, the handedness of a cryo-EM map for a β-sheet-only structure cannot be directly determined by visual inspection of the density.[11] Consequently, the model-building process was performed for both hands of the map to test which hand is more likely. The final handedness was selected based on which model yielded better refinement statistics, including clash scores, hydrogen bond formation, real-space correlation coefficients (RSCC), and Ramachandran favorite percentages. The hand of the map was thus finalized according to those statistics. Briefly, for each filament, models corresponding to the asymmetric unit (ASU) were first manually built and adjusted in Coot.[14] Subsequently, a filamentous model comprising multiple ASUs was generated in Chimera[15] to incorporate all necessary inter-ASU interfaces. This multimeric filament model was then real-space refined in PHENIX.[16] The final refinement and validation statistics are summarized in Table S1.

Western blot sample preparation

Cells were cultured to 80–90% confluency and lysed on ice with 500 µL lysis buffer supplemented with protease inhibitor cocktail per 10 cm dish. Lysates were sonicated for 10 s and subjected to three freeze–thaw cycles. After centrifugation at 15,294×g for 10 min at 4 °C, the supernatant was collected. For the supernatant fraction, proteins were denatured by adding sample loading buffer and heating at 95 °C for 5 min. Pellets were washed with lysis buffer, centrifuged again at 15,294×g for 10 min at 4 °C, resuspended in 50 µL sample loading buffer, heated at 95 °C for 5 min, and centrifuged at 15,294×g for 10 min at 4 °C to obtain the pellet fraction.

Lysate samples were loaded onto precast gels and run at 120 V for 45 min, then transferred to PVDF membranes at 100 V for 90 min on ice. Membranes were blocked in blocking buffer for 1 h at room temperature, incubated with primary antibodies (1:1,000) overnight at 4 °C, washed three times with TBST (TBS containing 0.1% Tween-20) for 5 min each, and incubated with secondary antibodies (1:5,000) for 1 h at room temperature. After six TBST washes, chemiluminescent substrate was applied for 1 min and membranes were scanned. For reprobing, membranes were stripped in stripping buffer for 20 min, washed with TBST, blocked for 1 h at room temperature, and incubated with the next primary antibody.

Actin polymerization kinetics

Actin Polymerization Buffer (10×) and ATP were thawed in a room temperature water bath, spun briefly to collect liquid, and kept on ice. G-buffer was prepared by supplementing General Actin Buffer with ATP (0.2 mM) and DTT (1 mM). Pyrene-labeled G-actin was diluted to 0.45 mg/mL with G-buffer, mixed gently,

and left on ice for 1 h to depolymerize preformed oligomers. Actin was centrifuged at 14,000 rpm at 4 °C for 30 min, and the supernatant transferred to a fresh tube on ice.

Peptide stock (20 mM) was prepared by dissolving peptide in PBS and adjusting the pH to 7.4 with 1 M NaOH. Pyrene-G-actin supernatant (200 µL per well) was dispensed into a glass-bottom 96-well plate: one well for actin monomer control, one for actin polymerization control, and the remaining wells for peptide at different concentrations. Peptide stock was added to the designated wells, mixed gently, and actin polymerization was initiated by adding 10× Actin Polymerization Buffer. Fluorescence was measured every 30 s for 1 h. The fluorescence baseline was subtracted for each sample, and the resulting time courses were fitted using a one-phase association model, $Y = Y_0 + (\text{Plateau} - Y_0)(1 - e^{-Kx})$, where Y_0 and Plateau represent the initial and equilibrium fluorescence levels, respectively, and K represents the apparent rate constant. The half-time was calculated as $t_{(1/2)} = \ln(2)/K$.

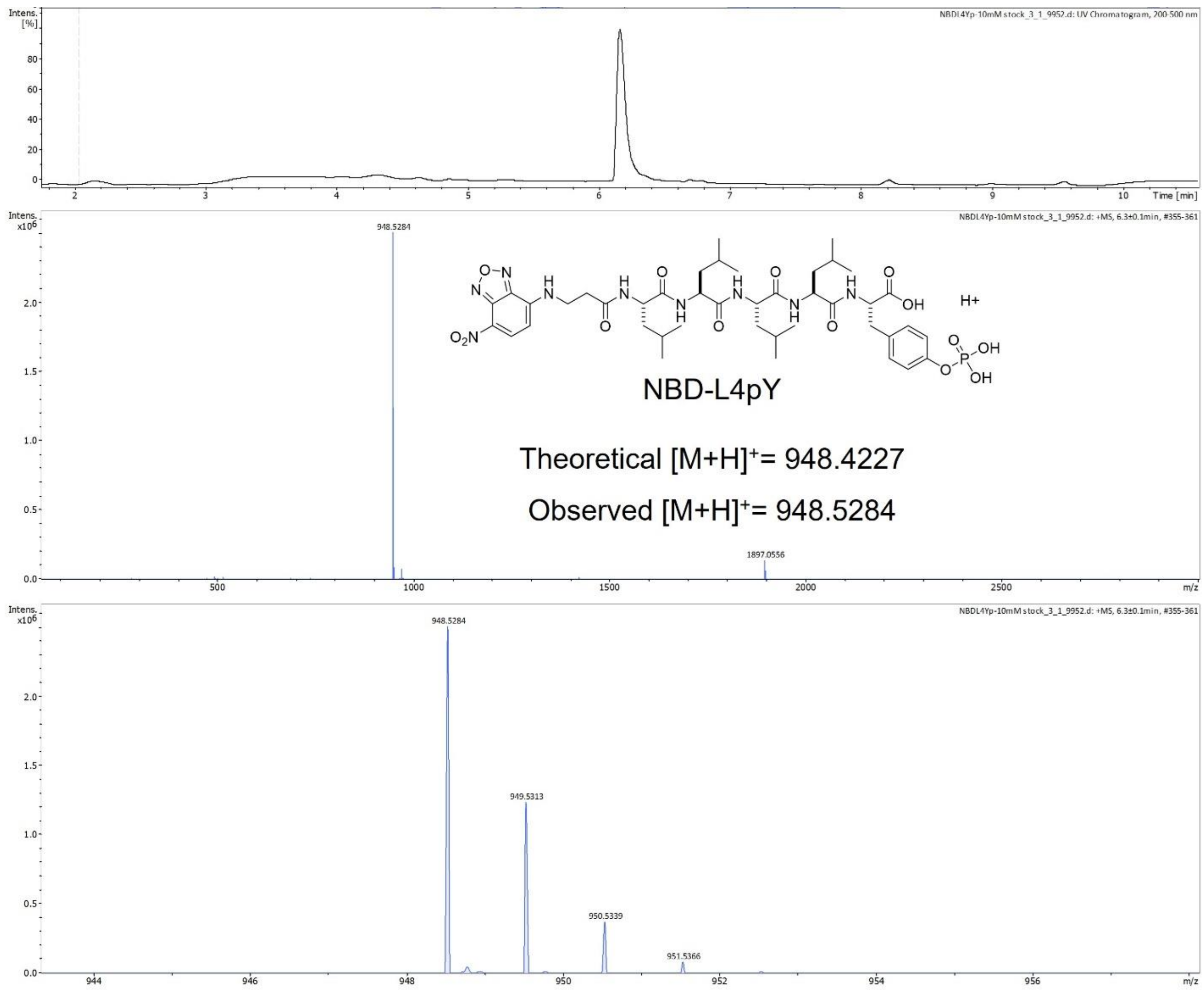

Figure S1. LC-MS spectra of NBD-L4$_p$Y.

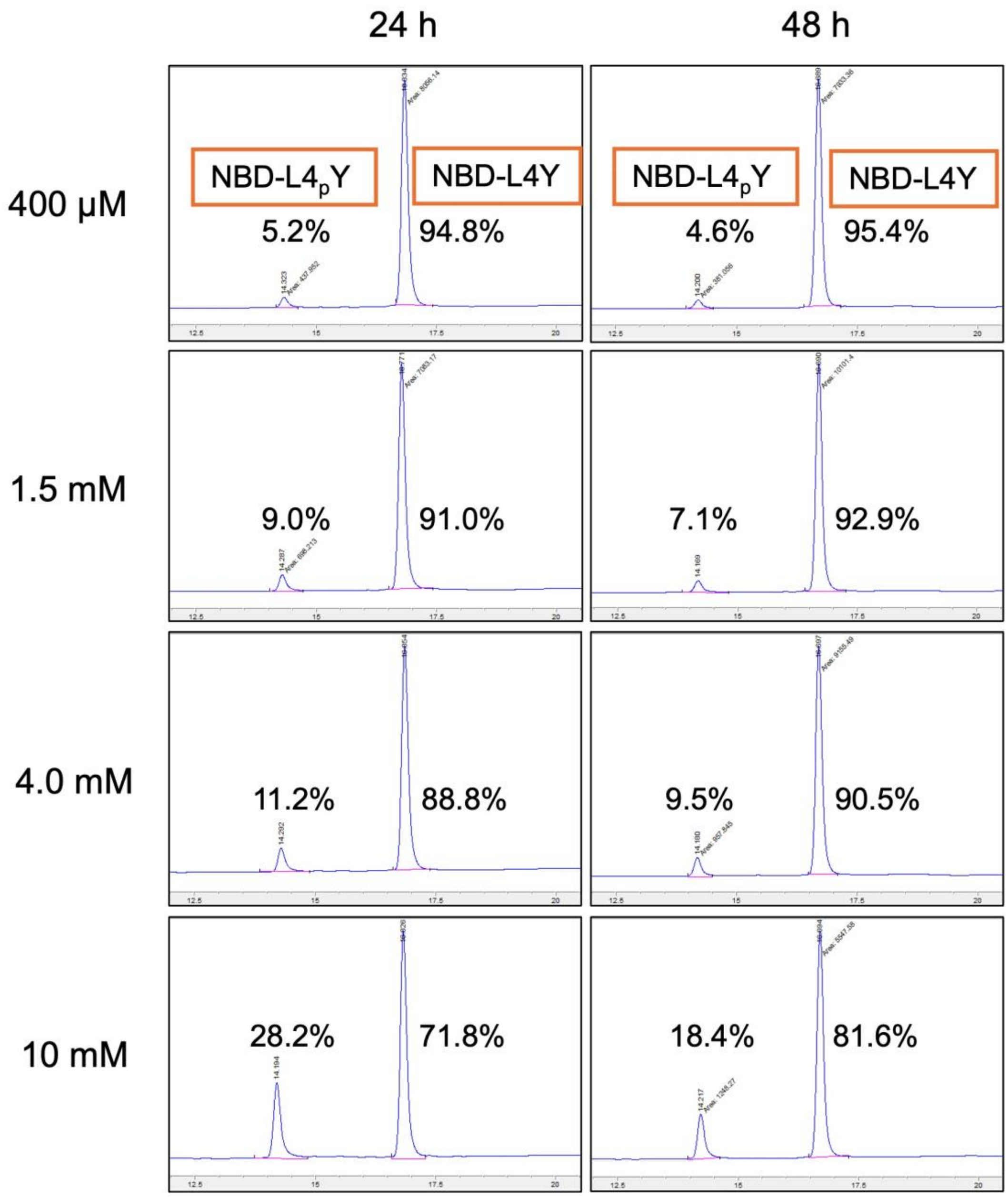

Figure S2. HPLC analysis of NBD-L4$_p$Y (0.40-10 mM) in the treatment of ALP (1 U/mL) for 24 or 48 hours.

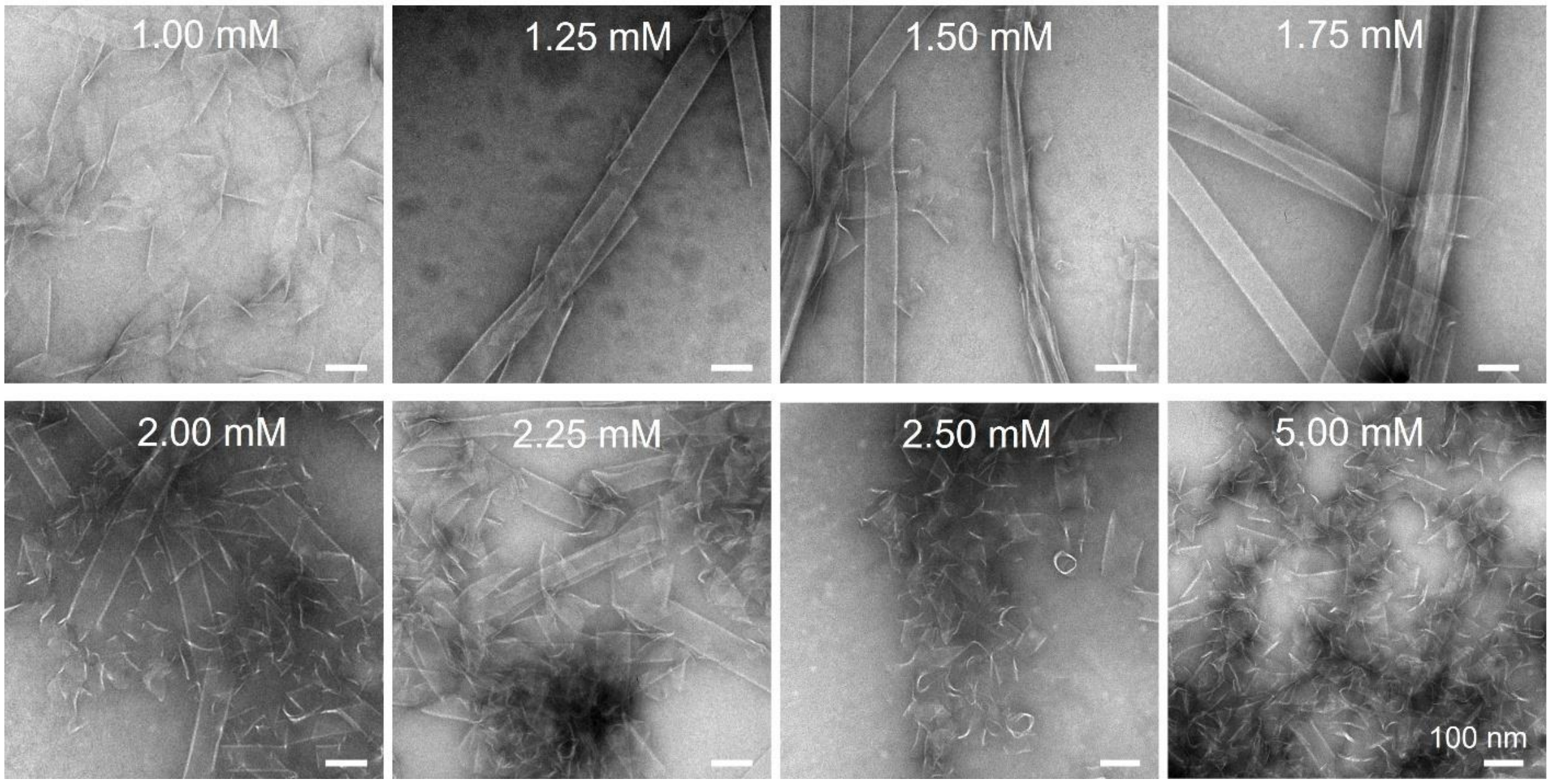

Figure S3. TEM images of (D)-NBD-l4$_p$y (1-5 mM) in the treatment of ALP (0.1 U/mL) for 24 hours.

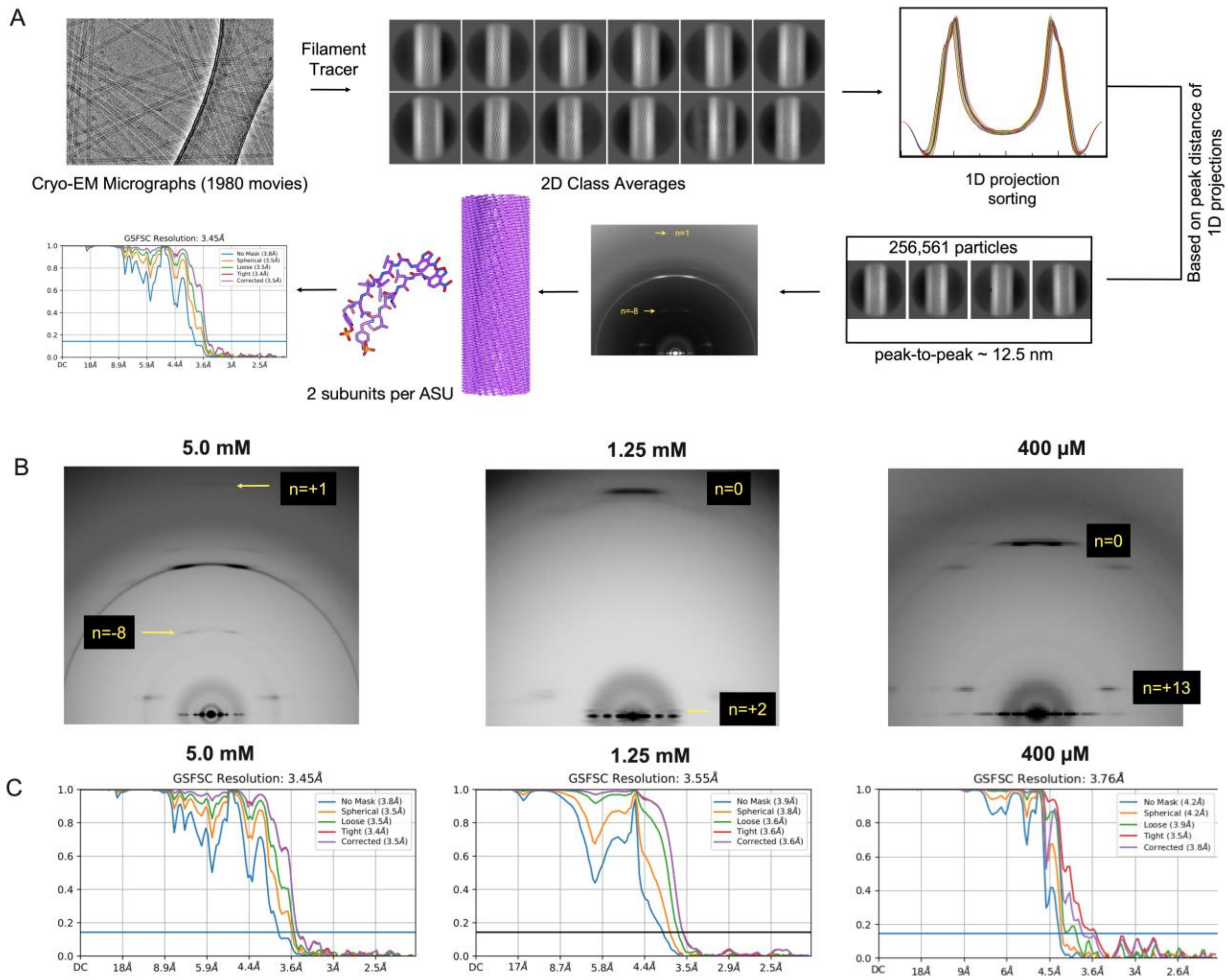

Figure S4. (A) Pipeline to sort out the filament tube diameter in Figure 1C. (B) Averaged power spectra of NBD-L4$_p$Y filaments prepared at distinct concentrations. (C) Fourier Shell Correlation (FSC) calculations of the NBD-L4$_p$Y filaments at distinct concentrations.

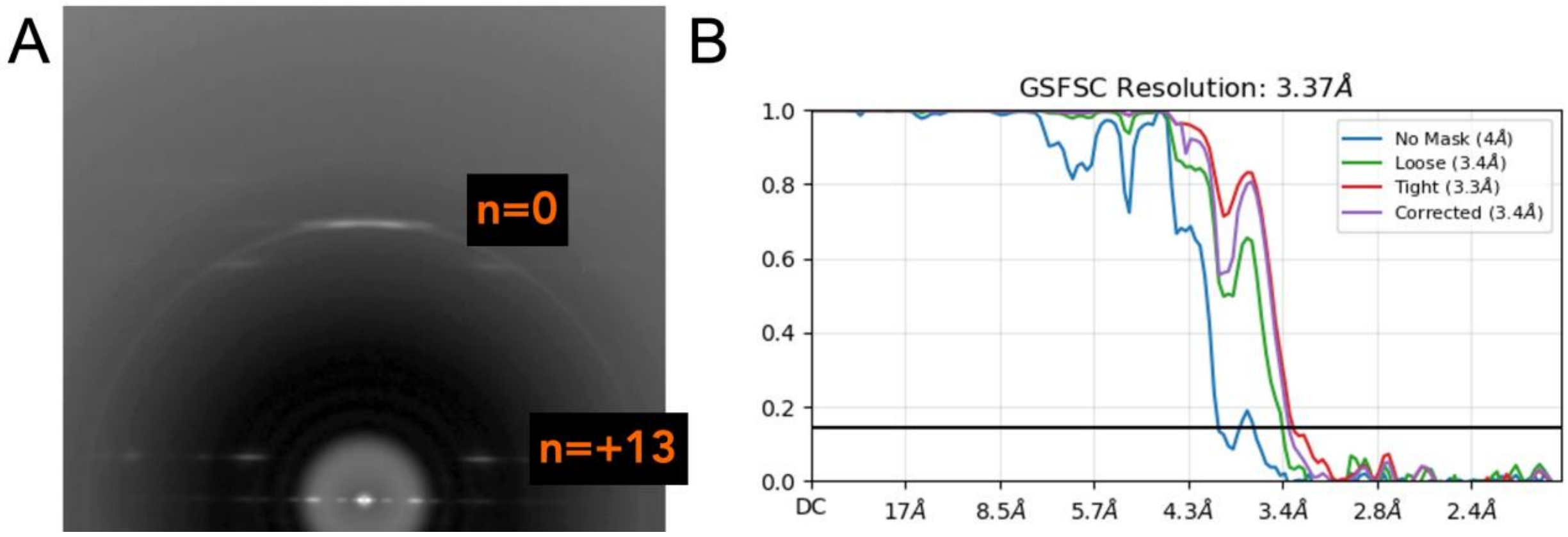

Figure S5. (A) Averaged power spectrum of *in situ* peptide filaments. (B) FSC calculations of the *in situ* NBD-L4$_p$Y filaments at 400 μM.

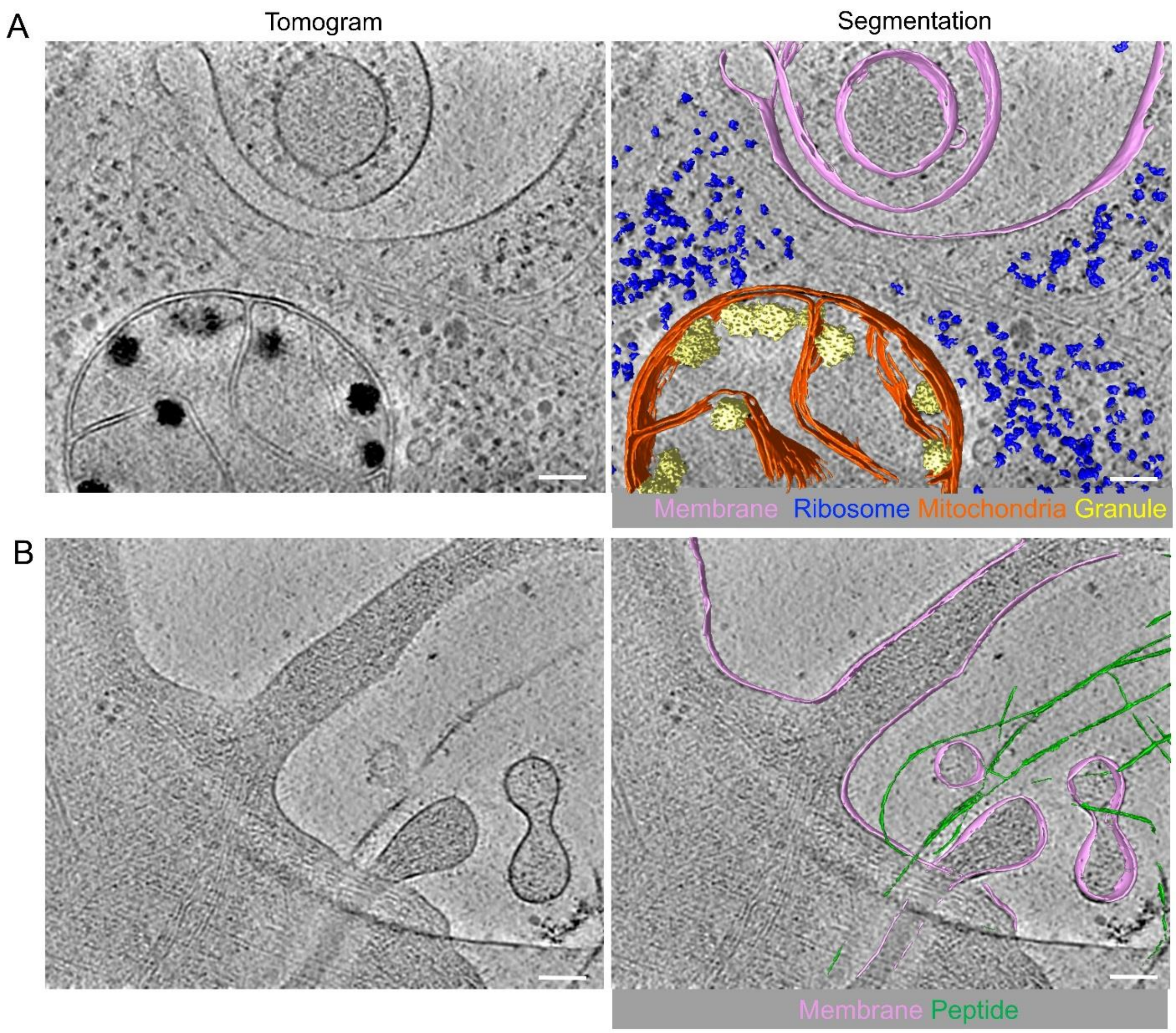

Figure S6. Tomograms and segmentations of Saos-2 cells incubated with NBD-L4$_p$Y (400 μM) for 2 minutes. Two field of views were displayed. Scale bar:100 nm.

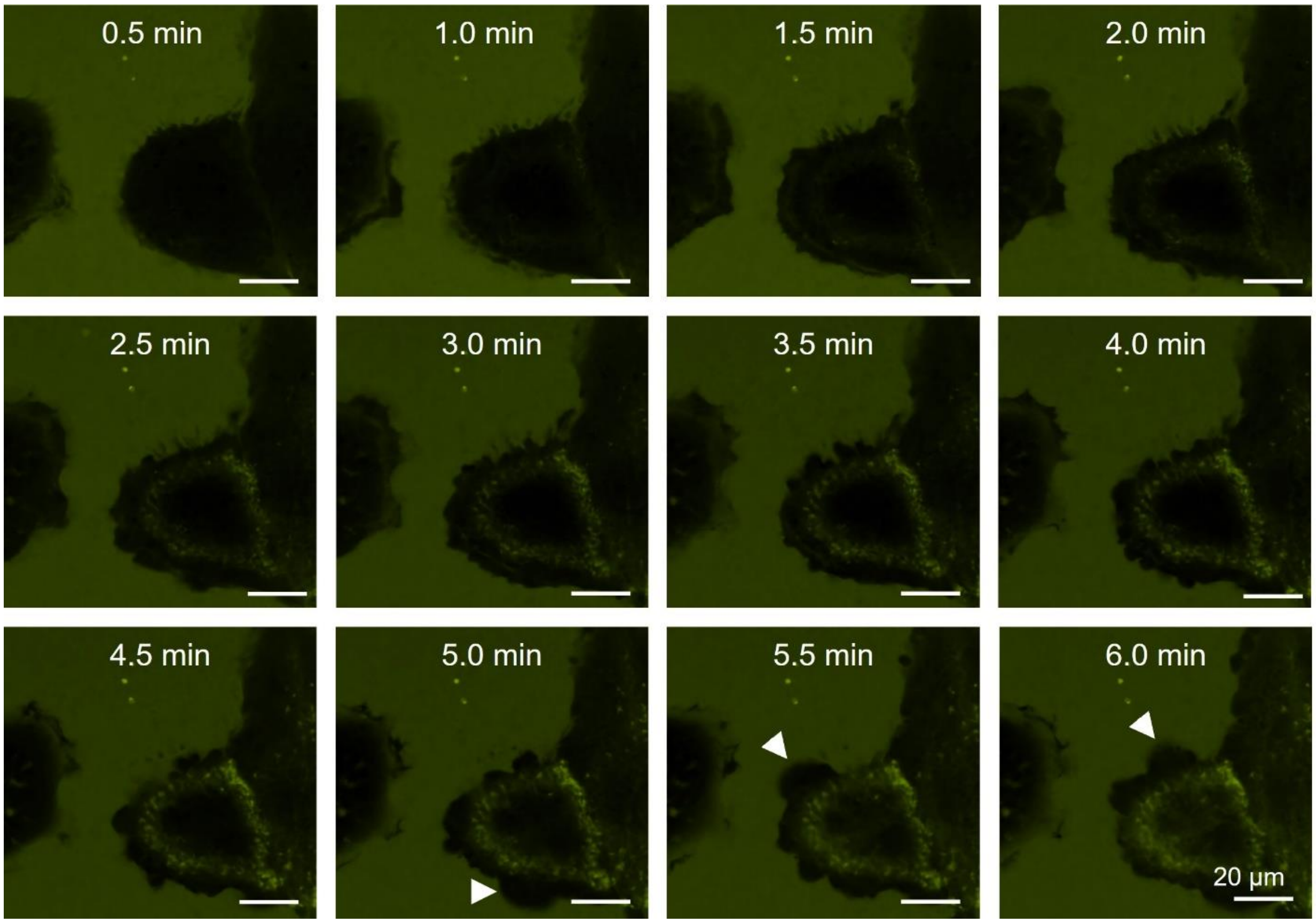

Figure S7. Live-cell confocal images of Saos-2 cells in the treatment of NBD-L4$_p$Y (400 µM) for the first 6 minutes. The arrowheads indicate membrane blebs.

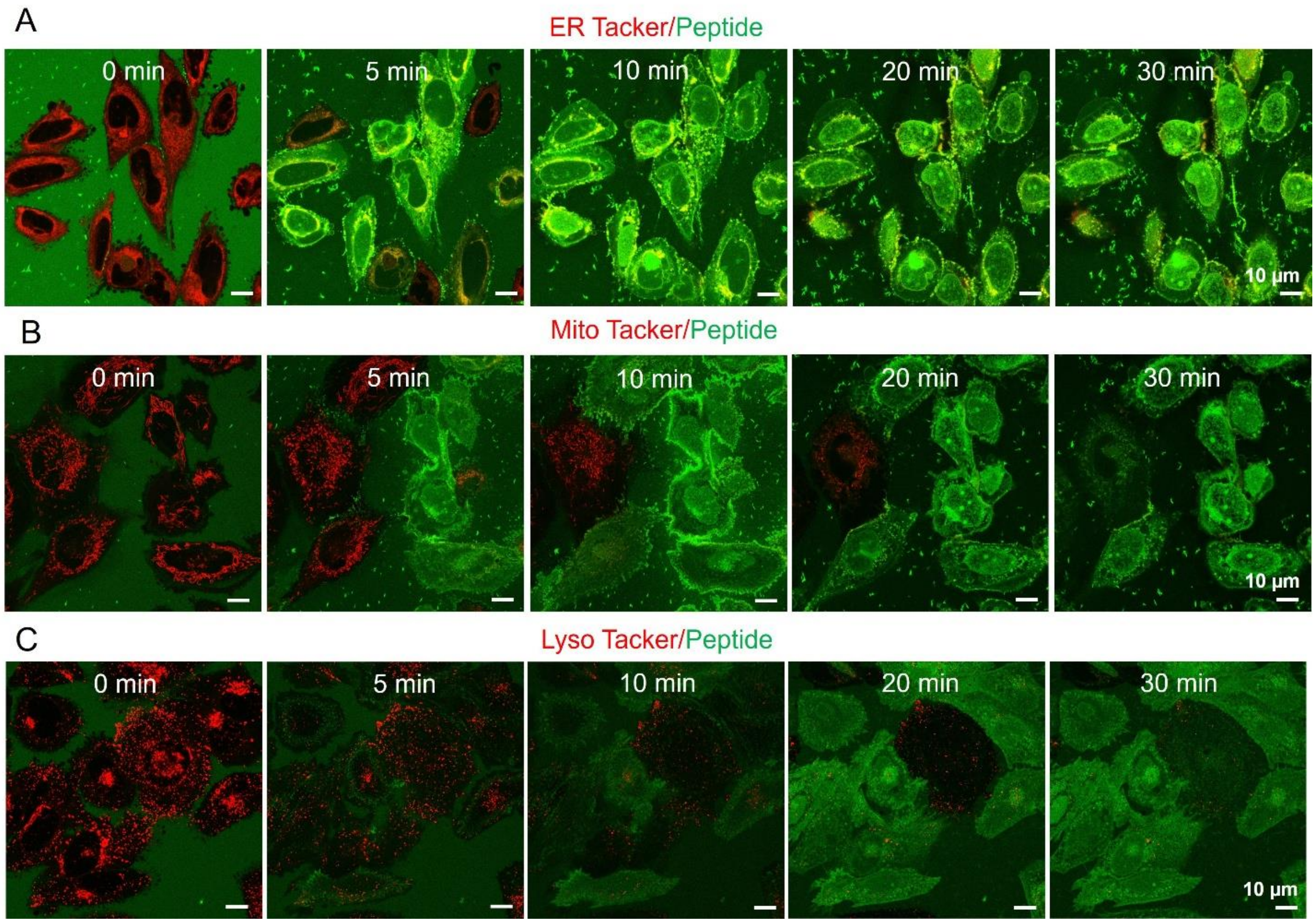

Figure S8. Live cell confocal imaging of Saos-2 cells incubated with NBD-$L4_pY$ (400 μM). Cells were pretreated with (A) ER Tracker, (B) Mito Tracker or (C) Lyso Tracker.

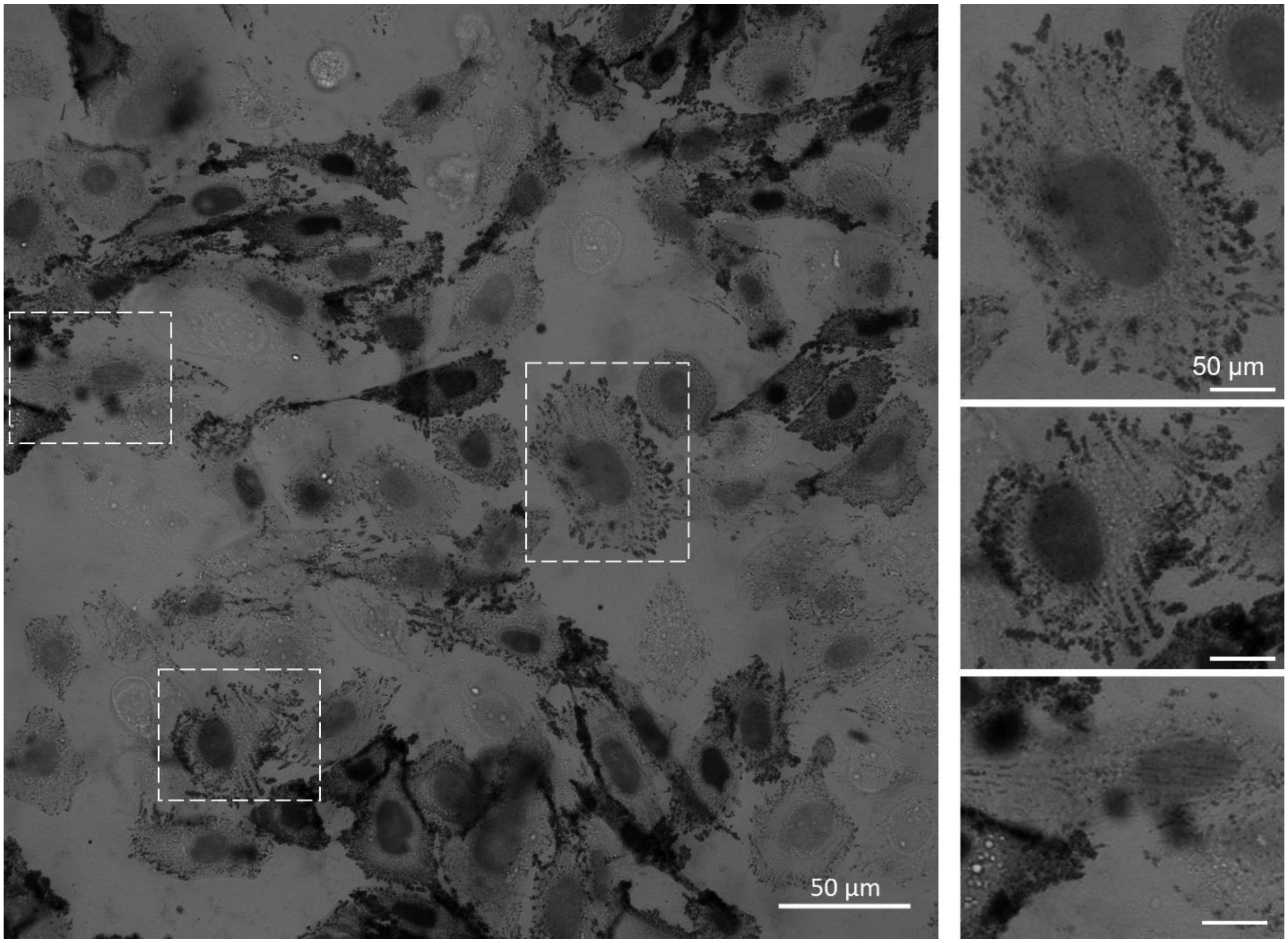

Figure S9. DIC images of Saos-2 cells incubated with NBD-L4$_p$Y (400 µM) for 30 minutes.

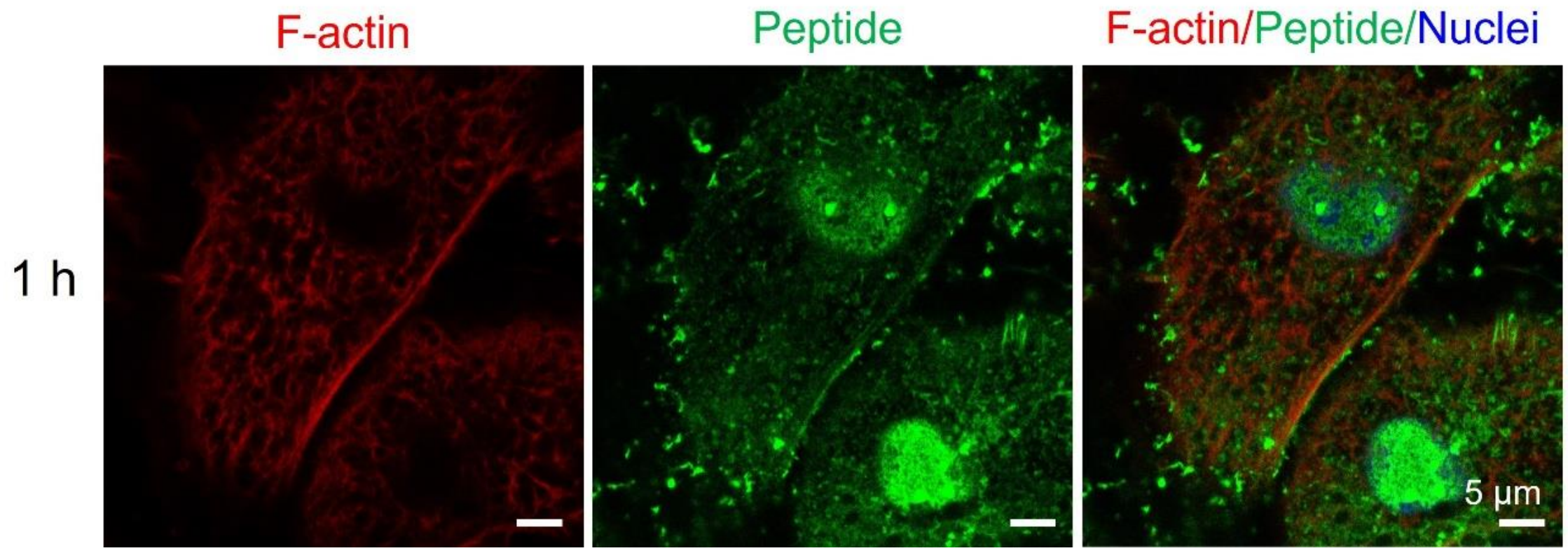

Figure S10. Confocal images of Saos-2 cells incubated with NBD-L4$_p$Y (400 μM) for 1 hour. Cells were fixed and stained with F-actin and Hoechst 33342 before imaging.

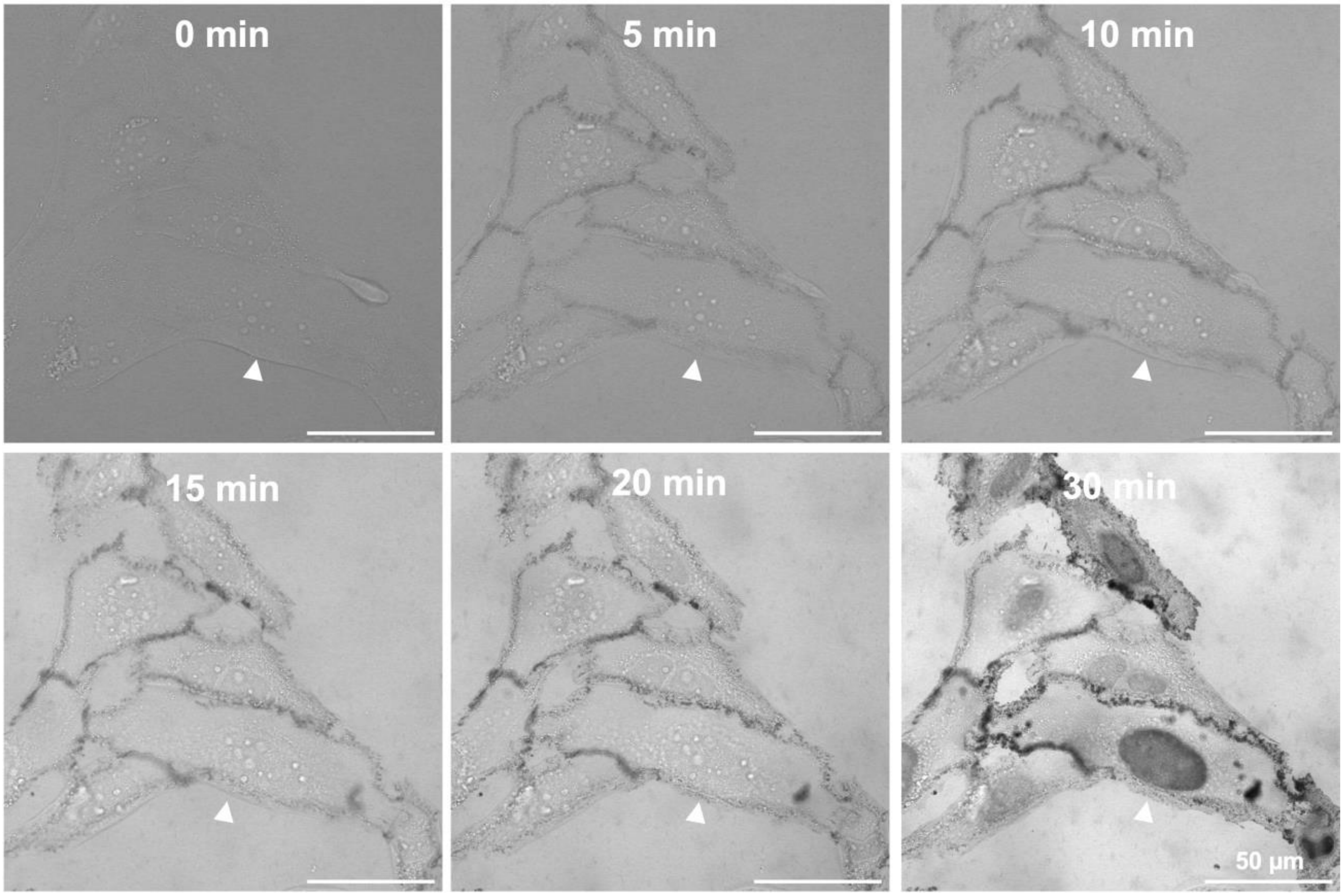

Figure S11. DIC images of Saos-2 cells incubated with NBD-L4$_p$Y (400 µM). Arrowheads indicate cell membrane.

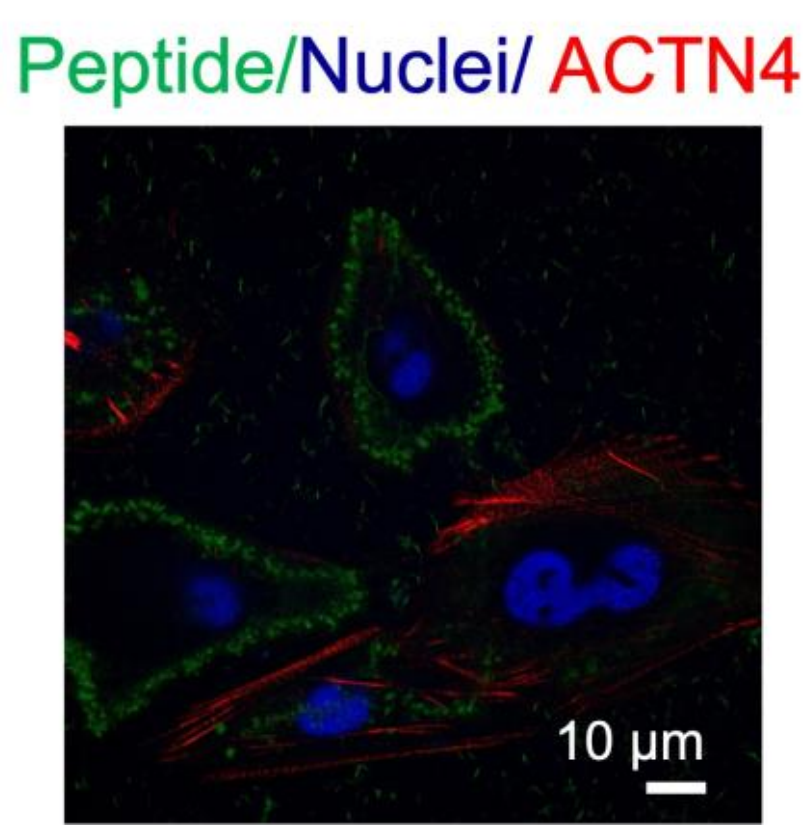

Figure S12. Immunofluorescence staining of ACTN4 in Saos-2 cells treated with NBD-L4$_p$Y for 15 min. [NBD-L4$_p$Y] = 400 µM.

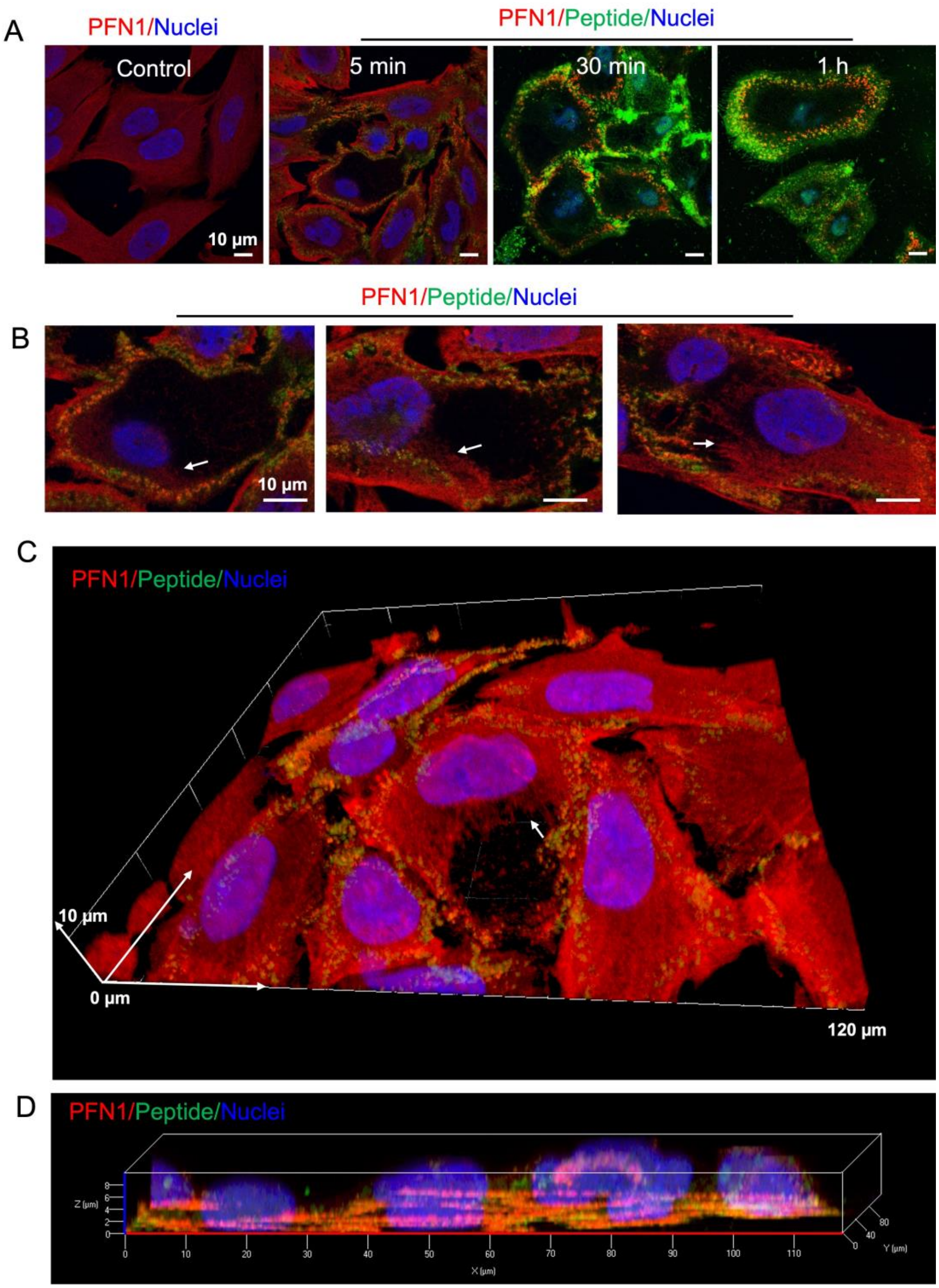

Figure S13. (A) Confocal images of Saos-2 cells incubated with NBD-L4$_p$Y (400 µM) for different durations. Cells were fixed and stained with PFN1 before imaging. (B) Zoom-in images of Saos-2 cells incubated with NBD-L4$_p$Y (400 µM) for 5 minutes. Arrows indicate regions with loss of cytoplasmic PFN1 signal associated with intracellular peptide assemblies. 3D rendering of Saos-2 cells incubated with NBD-L4$_p$Y (400 µM) for (C) 5 minutes or (D) 15 minutes.

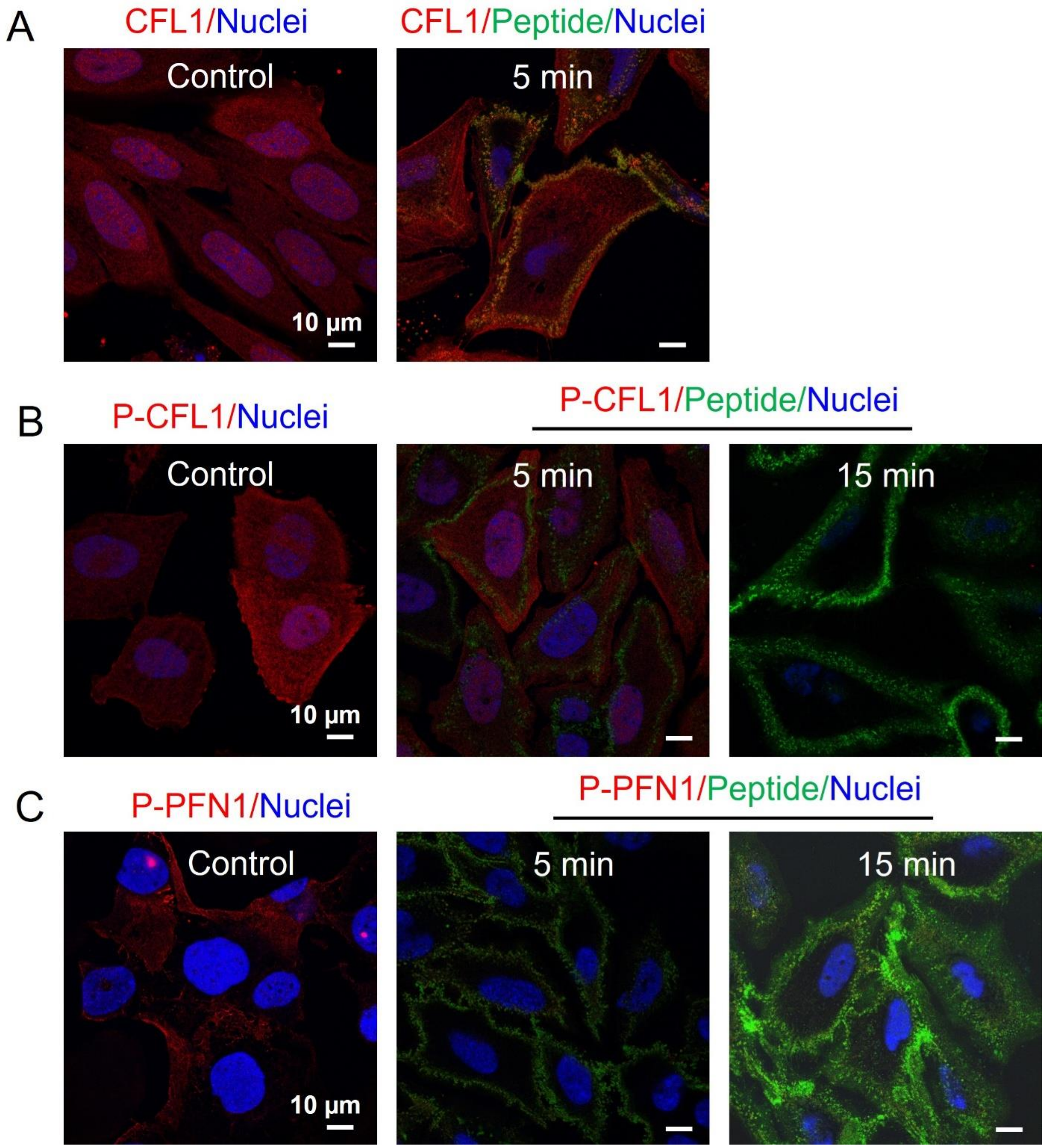

Figure S14. Confocal images of Saos-2 cells incubated with NBD-$L_4$$_p$Y (400 μM) for different durations. Cells were fixed and stained with (A) CFL1, (B) P-CFL1, or (C) P-PFN1 before imaging.

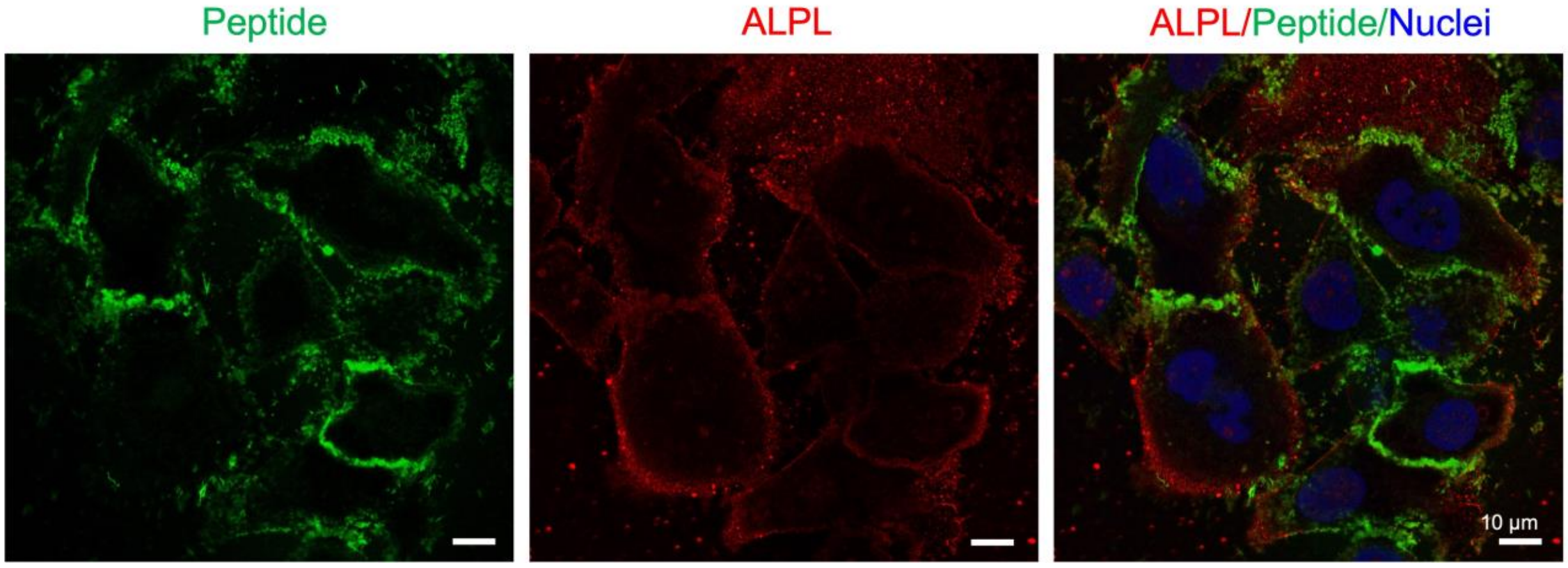

Figure S15. Confocal images of Saos-2 cells incubated with NBD-L4$_p$Y (400 µM) for 15 minutes. Cells were fixed and stained with ALPL before imaging.

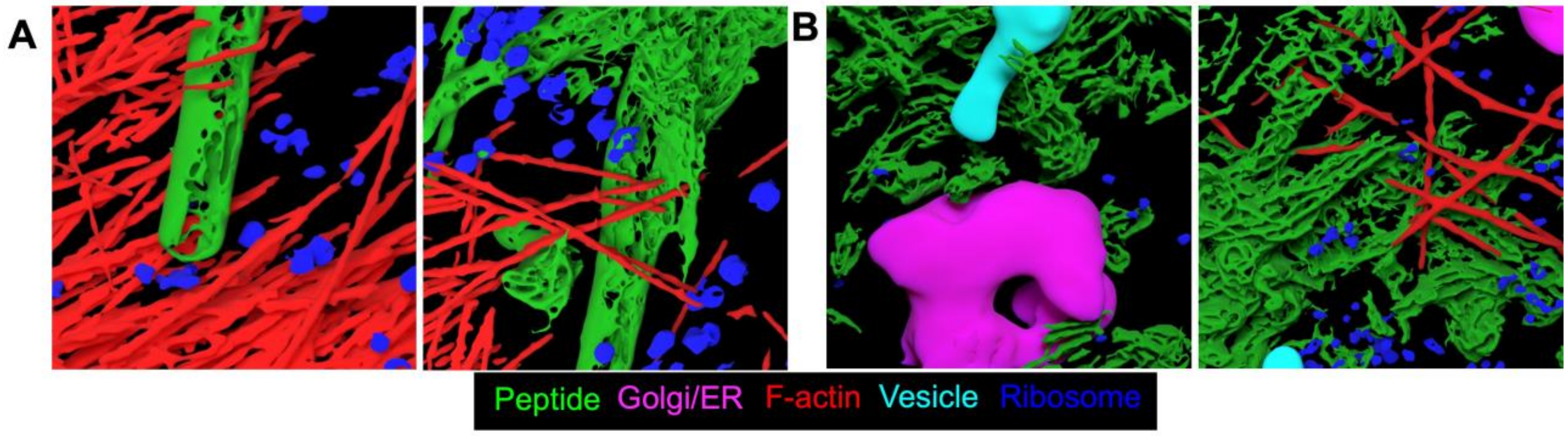

Figure S16. Segmentations of the boxed regions in Figure 5D (A) and Figure 5H (B) from cryo-ET tomograms of cryo-FIB–milled lamella of Saos-2 cells treated with NBD-L4pY for 15 min.

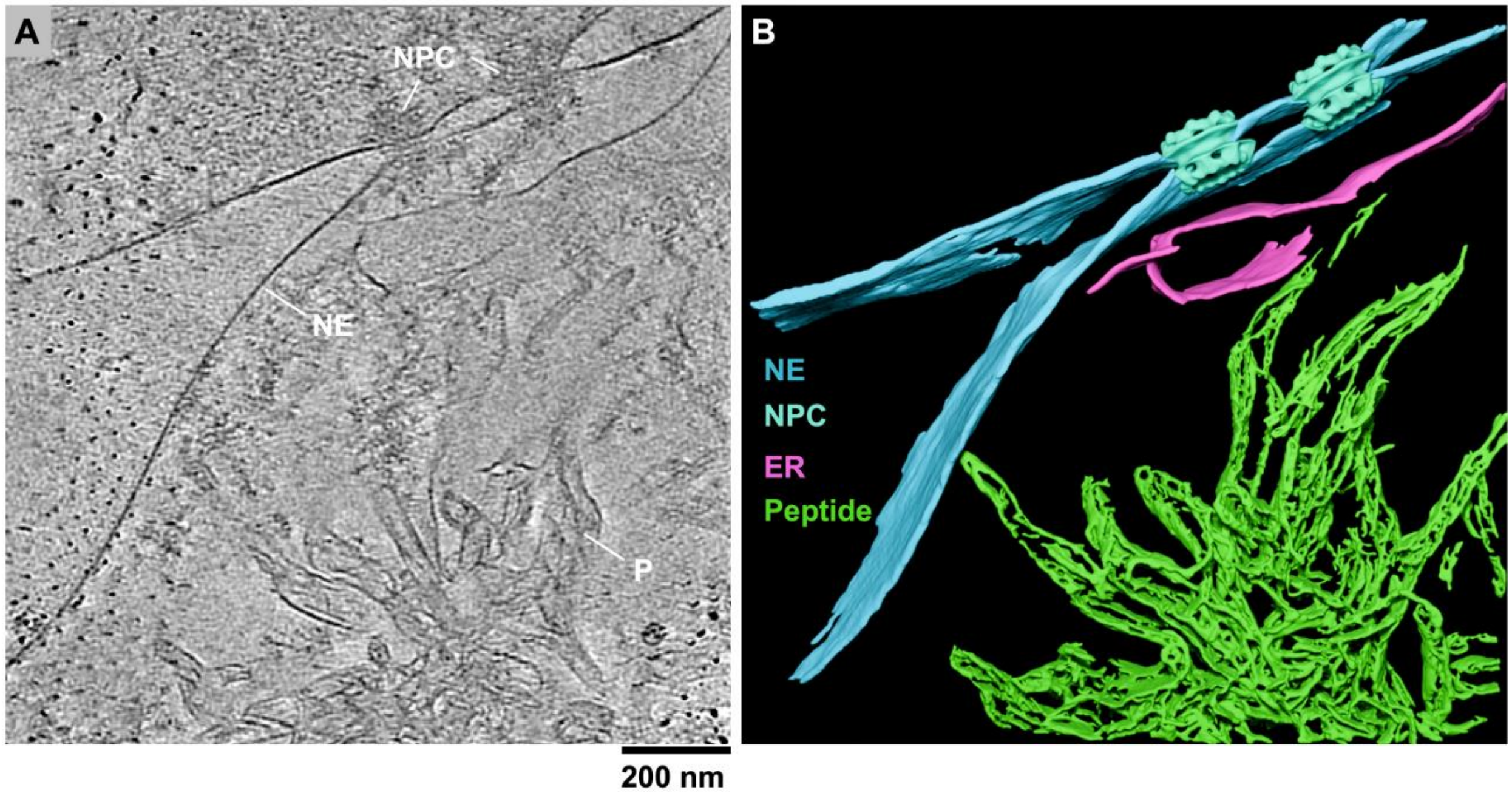

Figure S17. Intracellular peptide assemblies near the nuclear envelope in NBD-L4$_p$Y-treated Saos-2 cells. (A) Cryo-ET tomogram of a Saos-2 cell treated with 400 μM NBD-L4$_p$Y for 30 min, showing intracellular peptide assemblies (P) near the nuclear envelope (NE), nuclear pore complexes (NPC), and endoplasmic reticulum (ER). (B) Corresponding 3D segmentation showing the spatial organization between peptide assemblies and nuclear envelope.

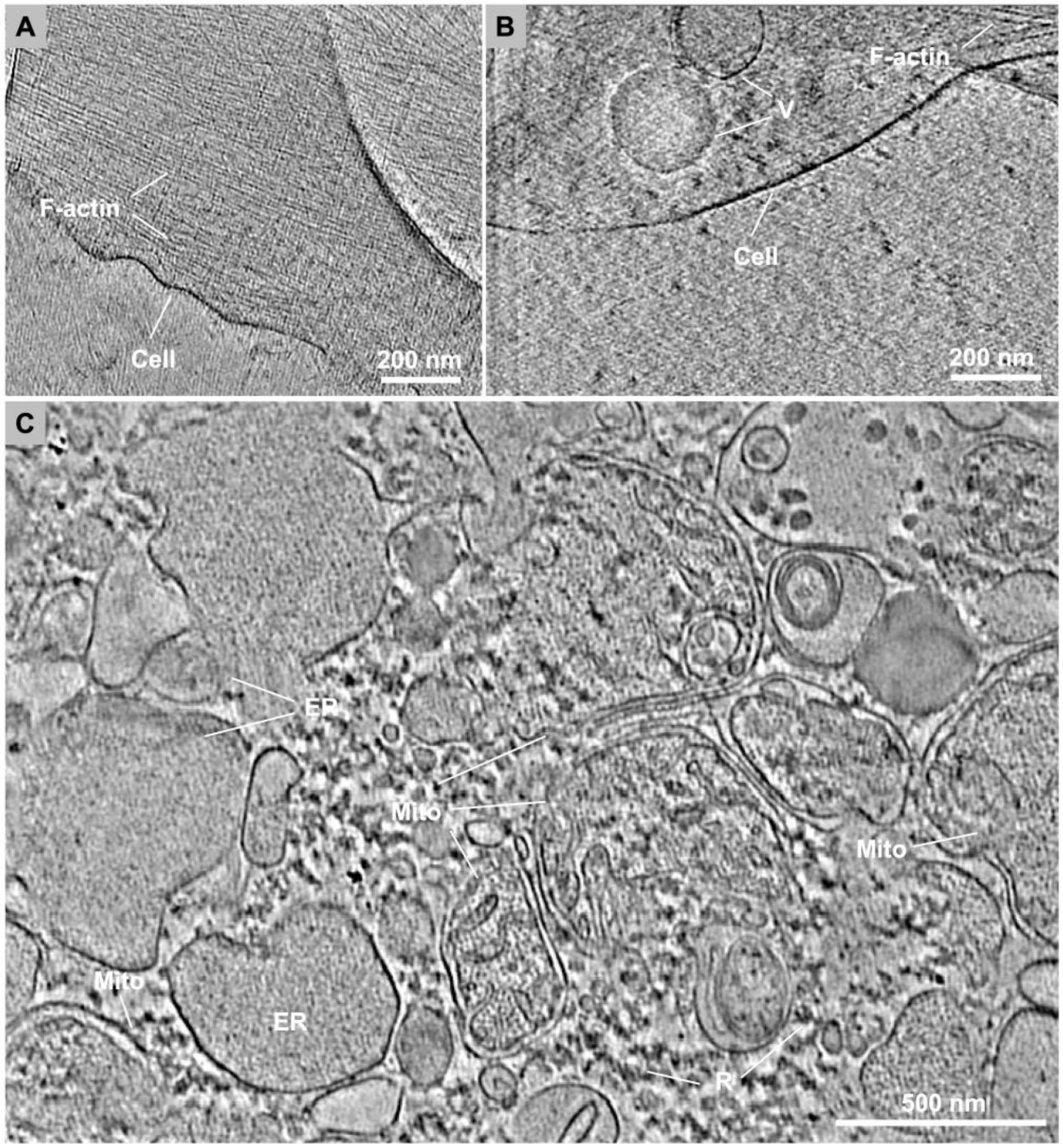

Figure S18. Control cryo-ET tomograms of Saos-2 cells. (A, B) Tomograms showing peripheral regions of untreated Saos-2 cells grown under control conditions and plunge-frozen for cryo-ET imaging. F-actin filaments, the cell boundary, and vesicles (V) are indicated. (C) Tomogram of a cryo-FIB-milled lamella showing the intracellular organization of a control Saos-2 cell, including mitochondria (Mito), endoplasmic reticulum (ER), and ribosomes (R).

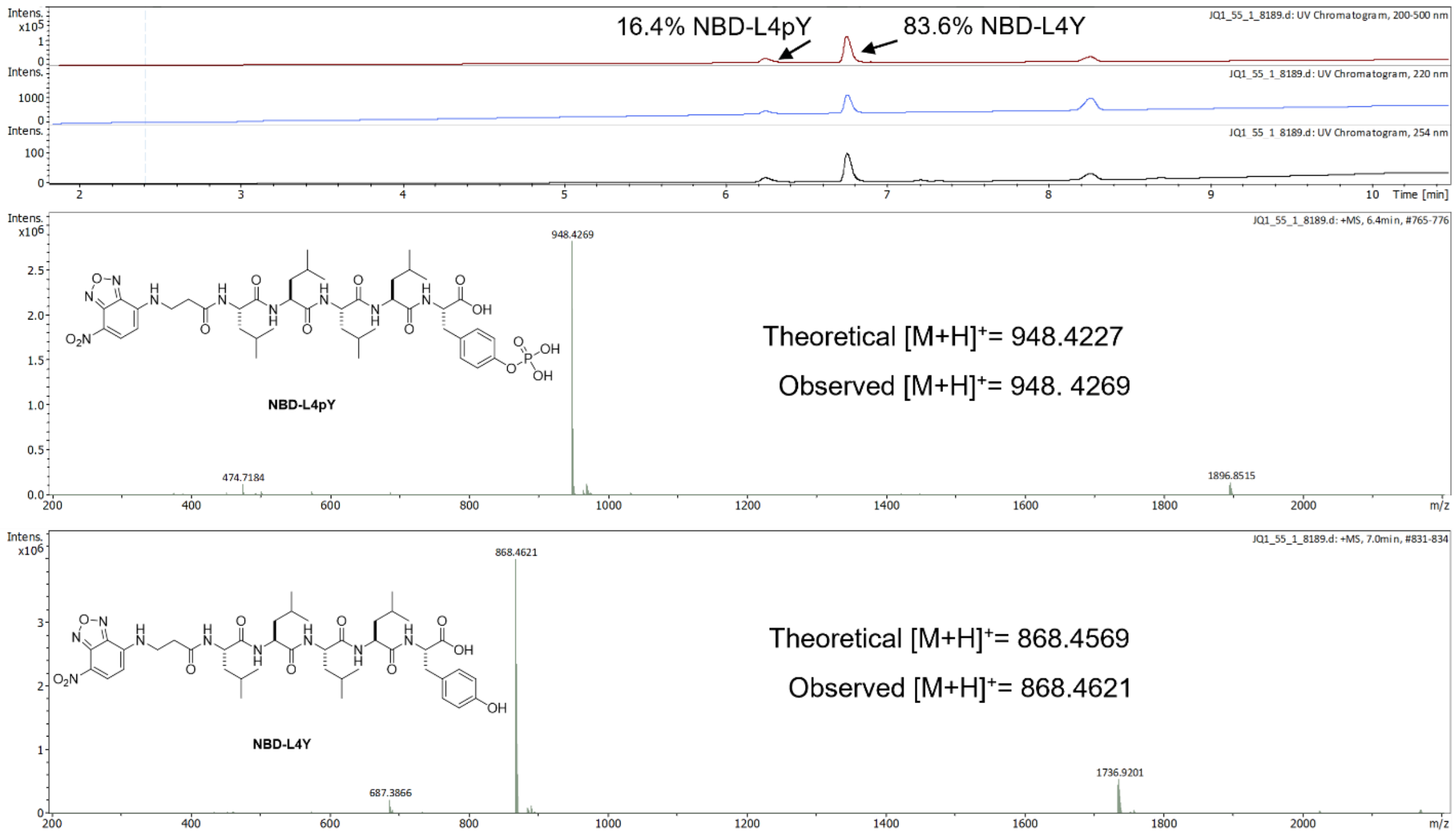

Figure S19. LC-MS spectra of the peptide that deposited on/into cells after 15 minutes of incubation with NBD-L4$_p$Y (400 μM).

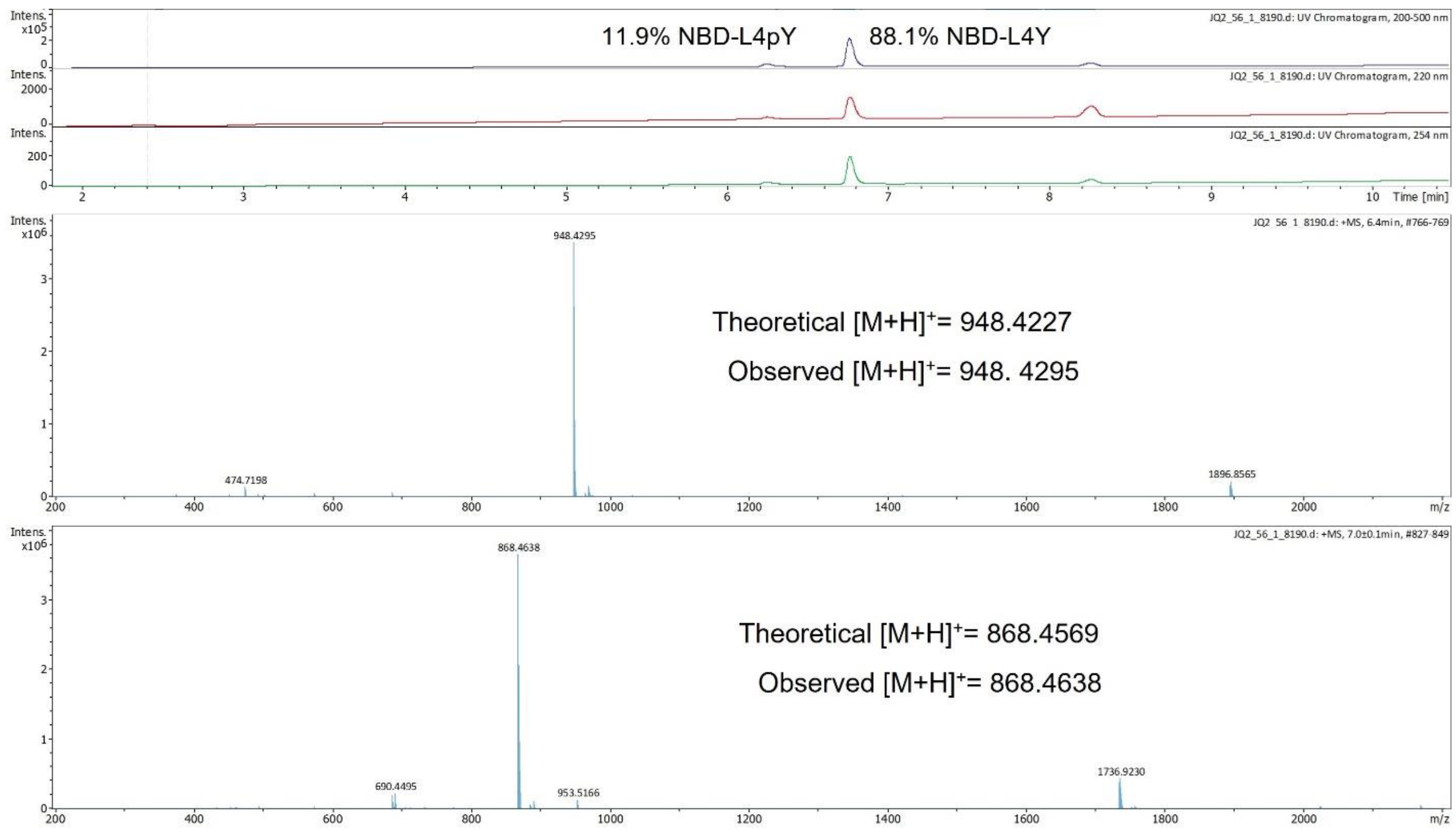

Figure S20. LC-MS spectra of the peptide that deposited on/into cells after 30 minutes of incubation with NBD-L4_pY (400 μM).

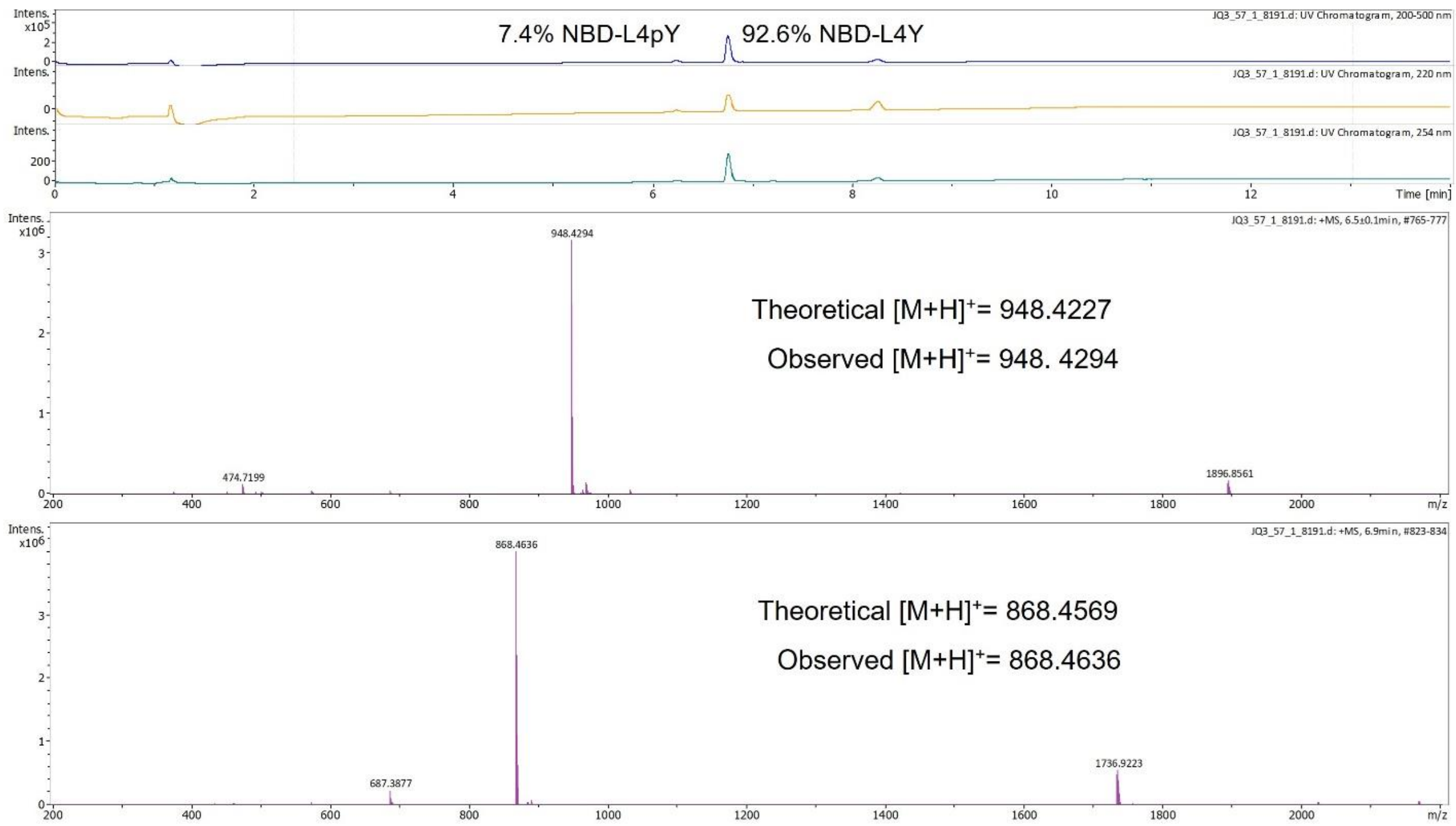

Figure S21. LC-MS spectra of the peptide that deposited on/into cells after 1 hour of incubation with NBD-$L4_pY$ (400 μM).

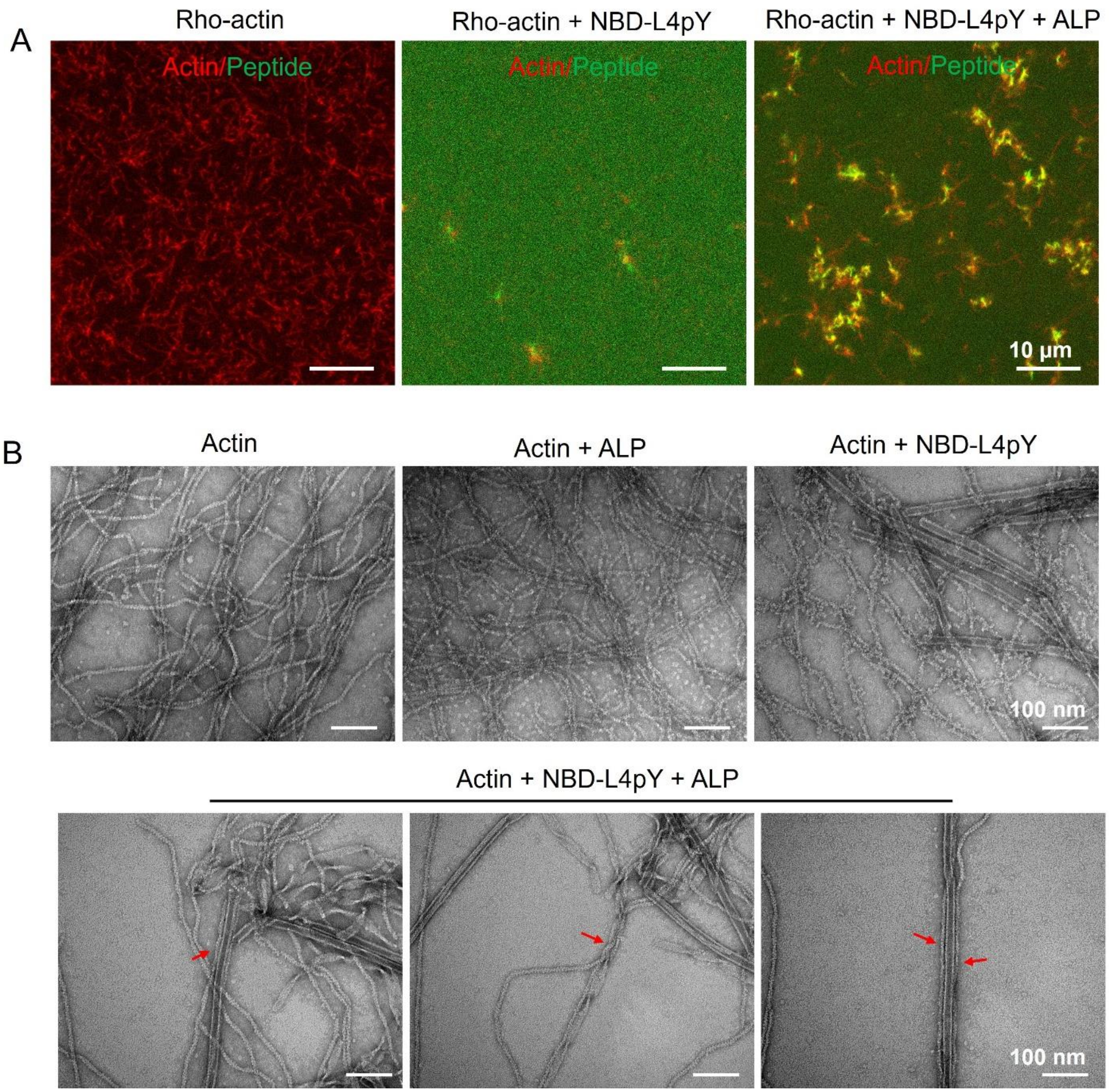

Figure S22. (A) Confocal images of rhodamine-actin (50 μg/mL) incubated in polymerization buffer for 3 hours and then treated with NBD-L4$_p$Y (400 μM), in the absence or presence of ALP (1 U/mL) for 24 hours. (B) TEM images of rhodamine-actin (200 μg/mL) incubated in polymerization buffer for 3 hours and then treated with ALP (1 U/mL), NBD-L4$_p$Y (400 μM), or both for 24 hours.

Table S1. Cryo-EM and refinement statistics of NBD-L4_pY at 5 mM, 1.25 mM, 0.4 mM, and *in situ* conditions.

**Parameters**	**NBD-L4_pY (5mM)**	**NBD-L4_pY (1.25mM)**	**NBD-L4_pY (0.4mM)**	**NBD-L4_pY (*In situ*)**
**Data collection and processing**				
Voltage (kV)	300	300	300	300
Electron exposure (e^{-} Å^{-2})	50	50	50	70
Pixel size (Å)	1.11	1.09	1.12	1.07
Particle images (n)	256,561	881,591	218,959	312,066
Shift (pixel)	10	10	10	20
**Helical symmetry**				
Point group	C1	C2	C13	C13
Helical rise (Å)	0.39	4.66	4.80	4.65
Helical twist (°)	43.070	4.01	4.05	4.17
**Map resolution (Å), Refinement and Model validation**				
Map:map FSC (0.143)	3.4	3.5	3.8	3.4
Clashscore	14	19.8	2.97	NA
Ramachandran Favored (%)	100%	98%	100%	NA
RSCC	0.71	0.84	0.70	NA
**Deposition ID**				
EMDB (Map)	EMD-49307	EMD-49330	EMD-49335	NA
PDB	9NEF	9NEQ	9NET	NA

References

1. Góngora-Benítez M, Tulla-Puche J, Albericio F. Handles for Fmoc Solid-Phase Synthesis of Protected Peptides. *ACS Combinatorial Science* **15**, 217-228 (2013).

2. Ottinger EA, Shekels LL, Bernlohr DA, Barany G. Synthesis of phosphotyrosine-containing peptides and their use as substrates for protein tyrosine phosphatases. *Biochemistry* **32**, 4354-4361 (1993).

3. Liu S*, et al.* Enzymatically Forming Intranuclear Peptide Assemblies for Selectively Killing Human Induced Pluripotent Stem Cells. *Journal of the American Chemical Society* **143**, 15852-15862 (2021).

4. Liu S*, et al.* Intranuclear Nanoribbons for Selective Killing of Osteosarcoma Cells. *Angewandte Chemie International Edition* **61**, e202210568 (2022).

5. Xu A, Xu C. FastTomo: A SerialEM Script for Collecting Electron Tomography Data. *bioRxiv*, 2021.2003.2016.435675 (2021).

6. Zheng SQ, Palovcak E, Armache J-P, Verba KA, Cheng Y, Agard DA. MotionCor2: anisotropic correction of beam-induced motion for improved cryo-electron microscopy. *Nature Methods* **14**, 331-332 (2017).

7. Kremer JR, Mastronarde DN, McIntosh JR. Computer Visualization of Three-Dimensional Image Data Using IMOD. *Journal of Structural Biology* **116**, 71-76 (1996).

8. Mastronarde DN, Held SR. Automated tilt series alignment and tomographic reconstruction in IMOD. *Journal of Structural Biology* **197**, 102-113 (2017).

9. Liu Y-T, Zhang H, Wang H, Tao C-L, Bi G-Q, Zhou ZH. Isotropic reconstruction for electron tomography with deep learning. *Nature Communications* **13**, 6482 (2022).

10. Punjani A, Rubinstein JL, Fleet DJ, Brubaker MA. cryoSPARC: algorithms for rapid unsupervised cryo-EM structure determination. *Nature Methods* **14**, 290-296 (2017).

11. Wang F, Gnewou O, Solemanifar A, Conticello VP, Egelman EH. Cryo-EM of Helical Polymers. *Chemical Reviews* **122**, 14055-14065 (2022).

12. Sanchez-Garcia R, Gomez-Blanco J, Cuervo A, Carazo JM, Sorzano COS, Vargas J. DeepEMhancer: a deep learning solution for cryo-EM volume post-processing. *Communications Biology* **4**, 874 (2021).

13. He J, Li T, Huang S-Y. Improvement of cryo-EM maps by simultaneous local and non-local deep learning. *Nature Communications* **14**, 3217 (2023).

14. Emsley P, Cowtan K. Coot: model-building tools for molecular graphics. *Acta Crystallographica Section D* **60**, 2126-2132 (2004).

15. Pettersen EF*, et al.* UCSF Chimera—A visualization system for exploratory research and analysis. *Journal of Computational Chemistry* **25**, 1605-1612 (2004).

16. Afonine PV*, et al.* Real-space refinement in PHENIX for cryo-EM and crystallography. *Acta Crystallographica Section D* **74**, 531-544 (2018).
